# Diet and colorectal cancer in UK Biobank: a prospective study

**DOI:** 10.1093/ije/dyz064

**Published:** 2019-04-17

**Authors:** Kathryn E Bradbury, Neil Murphy, Timothy J Key

**Affiliations:** 1 Cancer Epidemiology Unit, Nuffield Department of Population Health, University of Oxford, Oxford, UK; 2 National Institute for Health Innovation, School of Population Health, The University of Auckland, Auckland, New Zealand; 3 International Agency for Research on Cancer, World Health Organization, Lyon, France

**Keywords:** Diet, colorectal cancer, UK Biobank, red meat, processed meat, prospective study

## Abstract

**Background:**

Most of the previous studies on diet and colorectal cancer were based on diets consumed during the 1990s.

**Methods:**

We used Cox-regression models to estimate adjusted hazard ratios for colorectal cancer by dietary factors in the UK Biobank study. Men and women aged 40–69 years at recruitment (2006–10) reported their diet on a short food-frequency questionnaire (*n* = 475 581). Dietary intakes were re-measured in a large sub-sample (*n* = 175 402) who completed an online 24-hour dietary assessment during follow-up. Trends in risk across the baseline categories were calculated by assigning re-measured intakes to allow for measurement error and changes in intake over time.

**Results:**

During an average of 5.7 years of follow-up, 2609 cases of colorectal cancer occurred. Participants who reported consuming an average of 76 g/day of red and processed meat compared with 21 g/day had a 20% [95% confidence interval (CI): 4–37] higher risk of colorectal cancer. Participants in the highest fifth of intake of fibre from bread and breakfast cereals had a 14% (95% CI: 2–24) lower risk of colorectal cancer. Alcohol was associated with an 8% (95% CI: 4–12) higher risk per 10 g/day higher intake. Fish, poultry, cheese, fruit, vegetables, tea and coffee were not associated with colorectal-cancer risk.

**Conclusions:**

Consumption of red and processed meat at an average level of 76 g/d that meets the current UK government recommendation (≤90 g/day) was associated with an increased risk of colorectal cancer. Alcohol was also associated with an increased risk of colorectal cancer, whereas fibre from bread and breakfast cereals was associated with a reduced risk.

**Key Messages**

- Previous studies have found an increased risk of colorectal cancer in those with high intakes of red and processed meat. Most previous studies collected information on dietary intakes during the 1990s or earlier and patterns in meat consumption have since changed.
- In addition, few studies have used re-measured intakes to reduce the impact of measurement error, and to quantify the amount of red and processed meat that is associated with an increased risk. Measurement error generally biases the associations towards the null value; the associations observed in previous studies that did not re-measure intakes may be underestimated.
- Our study found that people who were consuming red and processed meat four or more times per week, had a 20% increased risk of colorectal cancer compared with those who were consuming red and processed meat less than twice a week.

## Background

In October 2015, the International Agency for Research on Cancer (IARC), part of the World Health Organization (WHO), concluded that there was sufficient evidence to classify processed meat as carcinogenic to humans (Group 1) and red meat as probably carcinogenic to humans (Group 2A).^1^ A recent systematic review of the published evidence on foods and beverages and colorectal cancer by the World Cancer Research Fund–American Institute for Cancer Research (WCRF-AICR) Continuous Update Project concluded there was convincing evidence that processed meat and alcoholic drinks increase the risk of colorectal cancer. The evidence that red meat increases the risk and that dairy products, wholegrains and foods containing dietary fibre reduce risk was judged as probable. The evidence for other foods and beverages was weak.^2^ Few studies included in the review corrected the results for measurement error and error in dietary assessment tends to attenuate the associations between diet and disease.^3^^,^^4^ Some studies included in the review did not publish results on all food types, which may indicate the presence of publication bias. In addition, many of the large previous studies were based on dietary intakes in the 1990s^5–7^ and food-supply data indicate that meat consumption has changed from the 1990s, with a much higher proportion of total meat supply in Europe and the USA now coming from poultry as opposed to beef and pork.^8^ Thus, it is not certain whether previous risk estimates are relevant to current eating patterns.

In addition, tumour characteristics^9^ as well as the underlying aetiology of bowel cancer^10^ may differ by anatomical sub-site, but there are few data on the associations between dietary factors and risk of sub-sites of colorectal cancer.

UK Biobank is a prospective cohort study of half a million men and women recruited between 2006 and 2010, and provides the opportunity to investigate prominent hypotheses related to diet and colorectal cancer in a contemporary population-based cohort in the UK. We have previously shown that the dietary data collected from the UK Biobank short food-frequency touchscreen questionnaire, which generally asks about frequency of consumption of main foods and food groups, is highly reproducible.^11^ These data are available on all UK Biobank participants. In addition, dietary intakes were re-measured in a large sub-sample of participants (*n* = 175 402) who completed at least one online 24-hour dietary assessment and these data can be used to correct for regression dilution and other forms of measurement error.^12^ The objective of the current study is to systematically examine the associations of colorectal-cancer risk with the intake of foods and food groups included in the short food-frequency touchscreen questionnaire: meat, fish, fruit, vegetables, milk, cheese, alcohol, tea and coffee, as well as fibre intake, and to use re-measured dietary intakes to quantify the risk at actual levels of intake in UK Biobank. We also examined the associations between intakes of food and food groups with anatomical sub-sites of colorectal cancer.

## Methods

### Study and participants

**Figure 1.**
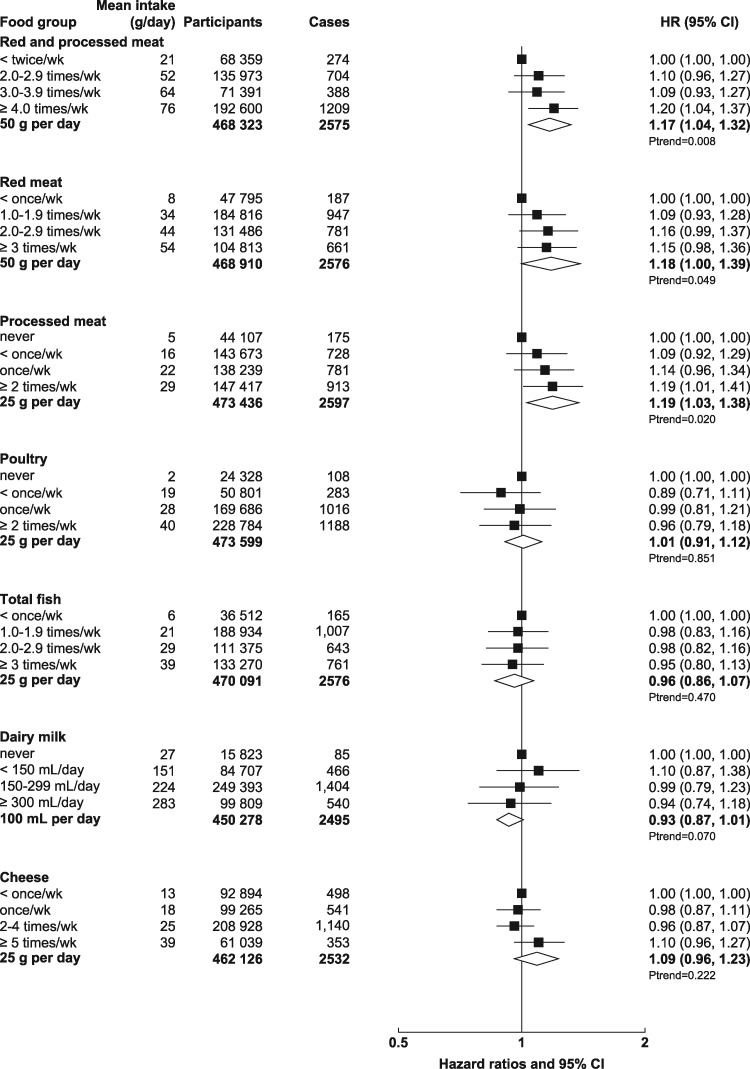
Hazard ratios (95% CIs) for the associations between animal foods and colorectal cancer in UK Biobank. Participants are categorized according to their intake at recruitment. Mean intake in each category is from the web-based 24-hour dietary assessments. Trend per increment uses the re-measured intakes from the web-based 24-hour dietary assessments. Models were stratified by age (<45, 45–, 50–, 55–, 60–, ≥65 years), sex, geographical region (10 regions) and socio-economic status (in fifths, based on the Townsend deprivation index^14^) and adjusted for education (College or University degree, vocational qualifications, optional national exams at ages 17–18 years, national exams at age 16 years, none of the above, unknown), smoking status (never, past, current <10 cigarettes per day, current 10–14 cigarettes per day, current 15–19 cigarettes per day, current 20+ cigarettes per day, unknown), waist circumference (sex-specific fifths, unknown), height (sex-specific fifths, unknown), physical activity [low: <10 excess metabolic equivalent (MET)-hours per week, moderate: 10–49.9 excess MET-hours per week, high: ≥50 excess MET-hours per week, unknown^15^], alcohol intake (<1, 1–7, 8–15, ≥16 g/day, unknown) (except for analyses where alcohol was the main exposure), family history of colorectal cancer (mother, father or sibling having had colorectal cancer, no family history, unknown), aspirin or ibuprofen use (regular use, not regular use, unknown), use of vitamin D supplements (regular use, not regular use, unknown), use of folate supplements (regular use, not regular use, unknown) and in women only: parity (0, 1–2, ≥3 live births, unknown), menopause (pre-menopausal, post-menopausal, unsure because of hysterectomy, unsure because of other reason, unknown), oral contraceptive agent (OCA) use (never, ever, unknown) and HRT use (never, ever, unknown).

**Figure 2.**
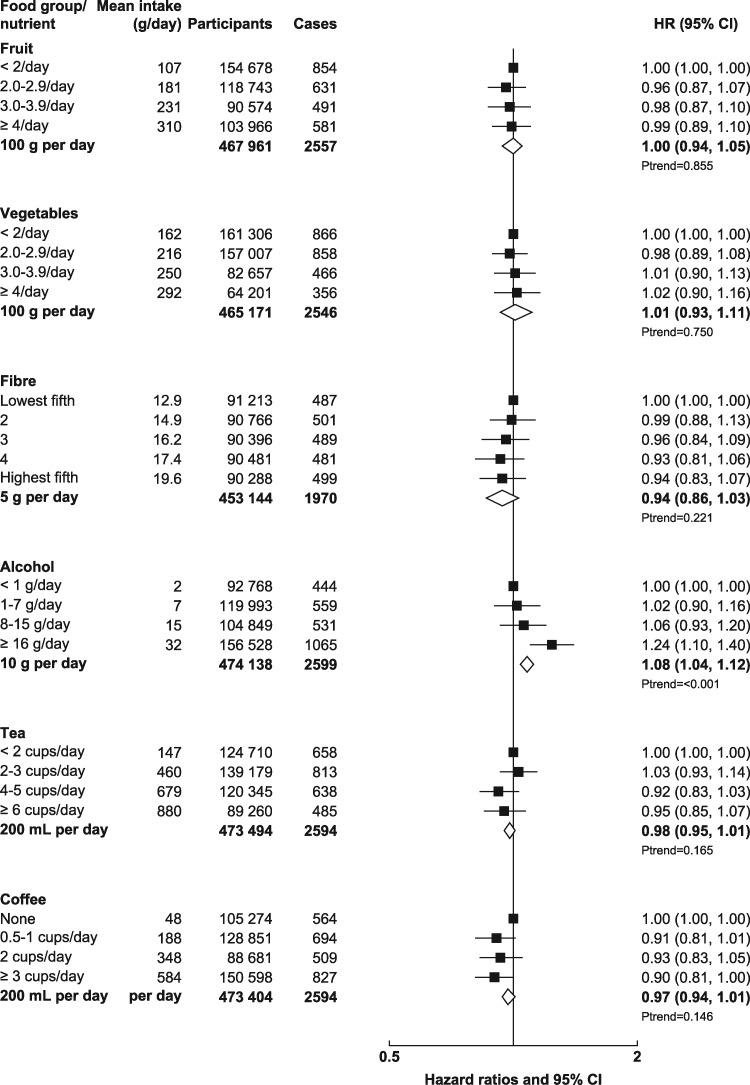
Hazard ratios (95% CIs) for the associations between plant foods, fibre, and alcohol and colorectal cancer in UK Biobank. Participants are categorized according to their intake at recruitment. Mean intake in each category is from the web-based 24-hour dietary assessments. Trend per increment uses the re-measured intakes from the web-based 24-hour dietary assessments. Models were stratified by age (<45, 45–, 50–, 55–, 60–, ≥65 years), sex, geographical region (10 regions) and socio-economic status (in fifths, based on the Townsend deprivation index^14^) and adjusted for education (College or University degree, vocational qualifications, optional national exams at ages 17–18 years, national exams at age 16 years, none of the above, unknown), smoking status (never, past, current <10 cigarettes per day, current 10–14 cigarettes per day, current 15–19 cigarettes per day, current 20+ cigarettes per day, unknown), waist circumference (sex-specific fifths, unknown), height (sex-specific fifths, unknown), physical activity [low: <10 excess metabolic equivalent (MET)-hours per week, moderate: 10–49.9 excess MET-hours per week, high: ≥50 excess MET-hours per week, unknown^15^], alcohol intake (<1, 1–7, 8–15, ≥16 g/day, unknown) (except for analyses where alcohol was the main exposure), family history of colorectal cancer (mother, father or sibling having had colorectal cancer, no family history, unknown), aspirin or ibuprofen use (regular use, not regular use, unknown), use of vitamin D supplements (regular use, not regular use, unknown), use of folate supplements (regular use, not regular use, unknown) and in women only: parity (0, 1–2, ≥3 live births, unknown), menopause (pre-menopausal, post-menopausal, unsure because of hysterectomy, unsure because of other reason, unknown), oral contraceptive agent (OCA) use (never, ever, unknown) and HRT use (never, ever, unknown).

UK Biobank is a prospective cohort study of half a million men and women aged 40–69 years recruited from across the UK between 2006 and 2010.^13^ The UK Biobank protocol is available online (http://www.ukbiobank.ac.uk/wp-content/uploads/2011/11/UK-Biobank-Protocol.pdf). Potential participants were identified from National Health Service patient registers and invited to attend a local assessment centre. Permission for access to patient records for recruitment was approved by the National Information Governance Board for Health and Social Care in England and Wales, and the Community Health Index Advisory Group in Scotland. At the assessment centre, the participants completed a touchscreen questionnaire, which collected information on socio-demographic characteristics and diet, lifestyle, reproductive and environmental factors. Anthropometric measurements were taken using standardized procedures. The touchscreen questionnaire and other resources are shown on the UK Biobank website (http://www.ukbiobank.ac.uk/resources/). Since recruitment, participants have been followed for cancer incidence and mortality via electronic linkage with cancer and death registries. UK Biobank has ethical approval from the North West Multi-centre Research Ethics Committee. At the touchscreen, all participants gave informed consent using a signature-capture device. The UK Biobank dataset for this project included 502 619 participants. We excluded participants with prevalent cancer [other than non-melanoma skin cancer, International Classification of Diseases, 10^th^ Revision (ICD-10) code C44] at recruitment (*n* = 27 038), leaving 475 581 participants (219 329 men and 256 252 women) included in the study. The number of participants in each analysis of food or nutrient and colorectal-cancer risk varies slightly, due to missing information for the exposure of interest [participants who selected ‘do not know’ or ‘prefer not to answer’ for the question(s) used to define the main exposure of interest]; the total numbers of participants for each exposure of interest are included in Figures 1 and 2.

### Assessment of dietary intakes

We have previously described the dietary data collected in UK Biobank.^11^ Briefly, at the recruitment assessment centre visit, participants completed a touchscreen questionnaire that included 29 questions on diet, most of which asked about the frequency of consumption of main foods and food groups. The questions used in this manuscript are those that asked about the frequency of consumption of processed meat, poultry, beef, lamb, pork, oily fish, non-oily fish, fresh fruit, dried fruit, raw vegetables, cooked vegetables, cheese, tea and coffee. A further 18 questions about alcohol consumption were used to estimate intakes of beer, wine (red and white) and spirits. More detail about the questions, and possible responses, is given in the Supplementary Methods, available as Supplementary data at *IJE* online. We also used the fibre score that we generated from the questions on fruit, vegetables, bread and breakfast cereal and described previously^11^ and which, when compared with the large sub-sample of participants who completed at least one web-based 24-hour dietary questionnaire, was shown to discriminate between people with low and high intakes of fibre.^11^ We also derived an estimate of dairy-milk intake, using the questions on consumption of main type of milk, bowls of breakfast cereal, cups of tea and cups of coffee. More details on the derivation of the estimate of milk intake are given in the Supplementary Methods, available as Supplementary data at *IJE* online. After recruitment, participants who had provided UK Biobank with an e-mail address were, between February 2011 and June 2012, invited via e-mail once every 3–4 months for a total of four times to complete an online 24-hour dietary assessment. A large sub-sample of participants (*n* = 175 402) completed at least one online 24-hour dietary assessment during this period.^14^ The 24-hour dietary assessment asks about the consumption of up to 206 types of foods and 32 types of drinks during the previous 24 hours.^15^ Portion sizes for each item were specified and participants select how many portions they consumed; for most items, a specific portion size was used, e.g. one apple, one sausage, one rasher of bacon, one slice of ham. For other items, such as pasta and rice, the portion size was specified as a ‘serving’. The mean daily intakes of nutrients were calculated by multiplying the frequency of consumption of each food or drink by a standard portion size and the nutrient composition of that particular item. The web-based 24-hour dietary assessment has been compared with an interviewer-administered 24-hour recall completed on the same day, with Spearman’s correlation coefficients for the majority of nutrients calculated from the web-based 24-hour dietary assessment ranging between 0.5 and 0.9 (median of 0.6).^15^

### Assessment of outcome

Prevalent and incident cancer cases within the UK Biobank cohort were identified through linkage to cancer and death registries. Eligible participants contributed person-years from date of attendance at the assessment centre at recruitment until the date of first registration with cancer [except non-melanoma skin cancer (ICD-10 C44)], date of death or last date of follow-up (30 November 2014 for England and Wales and 31 December 2014 for Scotland), whichever was earliest. The endpoint for these analyses is first diagnosis of colorectal cancer (ICD-10 codes C18–C20) or primary underlying cause of death colorectal cancer, whichever was first. Proximal colon cancers were defined as those that occurred within the caecum, appendix, ascending colon, hepatic flexure, transverse colon and splenic flexure (C18.0–C18.5). Distal colon cancers were those that occurred within the descending (C18.6) and sigmoid (C18.7) colon. Overlapping (C18.8) and unspecified (C18.9) lesions of the colon were categorized as colon cancers. Cancer of the rectum included cancers occurring at the recto sigmoid junction (C19) and rectum (C20). For participants who had a diagnosis of colon cancer and rectal cancer on the same date (*n* = 23) during follow-up (and no previous cancer diagnosis), colon cancer was assumed to be the first incident cancer in the sub-site analyses. For participants who had a diagnosis of proximal colon cancer and distal colon cancer on the same date (*n* = 10) during follow-up (and no previous cancer diagnosis), proximal colon cancer was assumed to be the first incident cancer in the sub-site analyses.

### Statistical analysis

We examined the associations between colorectal-cancer incidence and reported consumption of total red and processed meat, red meat, processed meat, poultry, fish, dairy milk, cheese, fruit, vegetables, fibre, alcohol, tea and coffee as reported on the short food-frequency touchscreen questionnaire at recruitment. Participants were generally divided into four categories of food intake according to their reported consumption on the short food-frequency touchscreen questionnaire at recruitment. The cut-offs for the categories were chosen so that there was a reasonable and similar number of participants in each group, as far as the distribution of data allowed. The mean food or nutrient intakes assigned to each baseline category were from the participants within each category who had completed at least one online 24-hour assessment (participants who had completed more than one 24-hour dietary assessment were assigned the mean from their records). These re-measured intakes were used to estimate the trends in risk; this approach reduces the impact of regression dilution bias and other forms of measurement error.^12^ We have previously described how we generated the mean intakes of foods and food groups from the 24-hour dietary assessments^11^; briefly, we grouped together foods in the 24-hour dietary assessment that corresponded to the same food group in the short food-frequency touchscreen questionnaire. For example, to get the mean intakes of non-oily fish, we calculated the sum (in grams) of the following items: white fish, battered fish, breaded fish and tinned tuna. For each participant who completed a 24-hour dietary assessment, the serving size in grams for each relevant food item was multiplied by the frequency reported in the 24-hour dietary assessment.^11^

Cox-regression models using attained age as the underlying time variable were used to estimate hazard ratios (HRs) for incident colorectal cancer by the reported frequency of foods, food groups and fibre from the short food-frequency touchscreen questionnaire at recruitment. Minimally adjusted analyses were stratified by age (<45 years, 45–, 50–, 55-, 60–, ≥65 years), sex, geographical region (10 regions) and socio-economic status (in fifths, based on the Townsend deprivation index^16^). For covariates where participants could select ‘do not know’ or ‘prefer not to answer’, these responses were combined into an ‘unknown’ category. Multivariable models were adjusted for education (College or University degree, vocational qualifications, optional national exams at ages 17–18 years, national exams at age 16 years, none of the above, unknown), smoking status (never, past, current <10 cigarettes per day, current 10–14 cigarettes per day, current 15–19 cigarettes per day, current 20+ cigarettes per day, unknown), waist circumference (sex-specific fifths, unknown), height (sex-specific fifths, unknown), physical activity [low: <10 excess metabolic equivalent (MET)-hours per week, moderate: 10–49.9 excess MET-hrs per week, high: ≥50 excess MET-hours per week, unknown^17^], alcohol intake (<1, 1–7, 8–15, ≥16 g/day, unknown) (except for analyses where alcohol was the main exposure), family history of colorectal cancer (mother, father or sibling having had colorectal cancer, no family history, unknown), aspirin or ibuprofen use (regular use, not regular use, unknown), use of vitamin D supplements (regular use, not regular use, unknown), use of folate supplements (regular use, not regular use, unknown) and in women only: parity (0, 1–2, ≥3 live births, unknown), menopause (pre-menopausal, post-menopausal, unsure because of hysterectomy, unsure because of other reason, unknown), oral contraceptive agent (OCA) use (never, ever, unknown) and hormone replacement therapy (HRT) use (never, ever, unknown).

We conducted a sensitivity analysis for colorectal cancer excluding participants who reported that they had made a major change in their diet in the past 5 years due to illness (*n* = 49 072) or preferred not to answer the question about whether they had made any major changes to their diet in the past 5 years (*n* = 1399). We also ran the fully adjusted model, adjusting for body mass index (BMI) (kg/m^2^: <20, 20–, 25–, 30–, unknown) instead of waist circumference. We also adjusted the result for total red and processed meat by each covariate separately, to examine which factors attenuated the minimally adjusted HR. To test for heterogeneity of the main associations by sex, we compared models with and without an interaction term for the main exposure (as the continuous trend variable) and sex and evaluated the significance using the likelihood ratio test. In an additional analysis, we further adjusted red meat for processed meat and vice versa using the fully adjusted model and fitting red meat and processed meat as continuous variables.

We investigated the association between incident colorectal cancer and type of fish (oily or non-oily) and type of alcoholic drink (beer, wine or spirits). We also investigated the association between incident colorectal cancer and source of fibre (fibre from fruit, vegetables or bread and breakfast cereals). For this analysis, the *P-*value for trend is based on the mean intakes at baseline in each category because estimates of fibre from fruit, vegetables and cereals are not available from the 24-hour dietary assessments.

For the main exposures, we also examined the associations for colon (ICD-10 code: C18) and rectal cancer (ICD-10 codes: C19–C20) separately and for proximal colon (ICD-10 codes: C18.0–C18.5) and distal colon (ICD-10 codes: C18.6–C18.7) separately. We also examined the associations between type of alcoholic drink and colon and rectal cancer separately because of the prior hypothesis that beer may increase the risk of rectal cancer.^18^^,^^19^ To test for heterogeneity of the main associations by sub-site (colon vs rectal and proximal colon vs distal colon), we fitted separate Cox-regression models for each sub-site using a competing-risk approach and compared the risk coefficients and standard errors in the subgroups of interest using likelihood ratio tests.^20^

Analyses were conducted in Stata version 14.1 (Stata Corp LP, College Station, TX). All *P-*values were two-sided.

## Results

Among 475 581 participants without prevalent cancer (excluding non-melanoma skin cancer) at recruitment, followed for an average of 5.7 years (maximum 8.6 years), 2609 cases of colorectal cancer occurred (1504 in men and 1105 in women). **Table 1** (men) and **Table 2** (women) compare participant characteristics between those in the lowest and highest categories of reported consumption of red meat, processed meat, poultry, fish, dairy milk and cheese. Compared with those in the lowest category, participants in the highest category of reported total red-meat intake were slightly older, more likely to be smokers, had a higher BMI and body-fat percentage, had a higher alcohol intake and had lower intakes of fruit, vegetables and fibre. The same was true for processed-meat intake, with the exception of age, which was similar between the two categories. Participants in the highest category of processed-meat intake (men and women combined) reported higher intakes of bacon, ham and sausages in the online 24-hour dietary assessments (mean daily intakes: 11.3 g of bacon, 7.5 g of ham and 6.2 g of sausages, total = 25.0 g per day) than those who reported never consuming processed meat at recruitment (mean daily intakes: 1.4 g of bacon, 1.2 g of ham and 0.8 g of sausages, total = 3.4 g per day) (data not shown). Supplementary Tables 1 (men) and 2 (women), available as Supplementary data at *IJE* online, compare participant characteristics between those in the lowest and highest categories of reported consumption of fruit, vegetables, total fibre, alcohol, tea and coffee.

**Table 1.** Association of lifestyle and anthropometric variables with intake of animal foods among men in UK Biobank

	Total red meat		Total processed meat		Poultry		Total fish		Dairy milk		Cheese	
	<1 time/wk	≥3 times/wk	Never	≥2 times/wk	Never	≥2 times/wk	<1 time/wk	≥3 times/wk	Never	≥300 mL/d	<1 time/wk	≥5 times/wk
*n*	15 789	55 388	11 875	94 711	8525	104 407	16 709	59 275	6857	50 207	37 291	29 975
Mean (SD) age, years	54.9 (8.3)	57.0 (8.1)	55.6 (8.3)	56.1 (8.3)	55.1 (8.3)	55.6 (8.3)	53.9 (8.2)	57.5 (8.1)	56.3 (8.1)	57.1 (8.0)	56.7 (8.1)	56.0 (8.2)
% (*n*) highest fifth of socio-economic status^a^	14.7 (2310)	19.6 (10856)	14.9 (1767)	19.5 (18405)	13.8 (1177)	21.0 (21920)	15.6 (2599)	20.0 (11850)	15.6 (1068)	20.6 (10200)	16.9 (6309)	21.3 (6366)
% (*n*) current smokers	10.5 (1664)	15.5 (8577)	9.9 (1170)	15.1 (14340)	12.3 (1046)	11.6 (12 098)	16.6 (2773)	11.6 (6853)	17.0 (1163)	15.1 (7592)	12.6 (4697)	12.5 (3759)
Mean (SD) height, cm	175.3 (7.0)	175.4 (6.9)	175.1 (7.2)	175.7 (6.9)	175.5 (7.2)	175.8 (6.8)	175.2 (7.1)	175.5 (6.8)	175.0 (6.9)	175.8 (6.8)	174.4 (6.8)	177.0 (6.8)
Mean (SD) BMI, kg/m^2^	26.6 (4.1)	28.4 (4.4)	26.3 (3.9)	28.2 (4.4)	26.4 (4.3)	28.2 (4.3)	27.9 (4.6)	27.8 (4.3)	28.0 (4.7)	27.8 (4.2)	28.1 (4.4)	27.3 (4.3)
Mean (SD) waist circumference, cm	93.7 (11.3)	98.3 (11.7)	92.9 (10.9)	98.0 (11.7)	93.7 (11.6)	97.3 (11.3)	97.2 (12.0)	96.6 (11.4)	97.3 (12.3)	96.9 (11.4)	97.1 (11.6)	96.1 (11.5)
Mean (SD) body fat, %	23.7 (6.0)	25.9 (5.8)	23.3 (6.0)	25.6 (5.9)	23.5 (6.3)	25.5 (5.7)	25.4 (6.0)	25.2 (5.8)	25.6 (6.1)	25.0 (5.8)	25.7 (5.9)	24.2 (6.0)
% (*n*) family history of CRC^b^	7.6 (1203)	9.0 (5000)	7.6 (901)	8.9 (8383)	7.7 (656)	8.6 (8952)	7.7 (1280)	8.8 (5233)	8.2 (563)	9.1 (4543)	8.2 (3049)	8.8 (2640)
% (*n*) regular NSAID use^c^	25.6 (4040)	29.8 (16 496)	25.6 (3034)	28.9 (27 398)	23.6 (2014)	29.2 (30 490)	26.7 (4459)	30.7 (18 223)	28.7 (1967)	28.7 (1967)	31.1 (11 595)	26.3 (7879)
Mean (SD) alcohol, g per day	19.7 (22.0)	28.7 (26.7)	19.0 (19.7)	27.3 (25.7)	21.3 (24.5)	24.7 (22.8)	23.5 (27.2)	25.1 (22.8)	31.1 (30.8)	21.7 (21.8)	22.7 (23.5)	25.1 (23.8)
Mean red meat, g per day^d^	9	59	11	49	8	45	35	39	42	43	43	39
Mean processed meat, g per day^d^	9	29	4	32	4	27	23	22	26	24	24	23
Mean fruit, g per day^d^	205	170	221	161	202	178	159	204	184	189	195	177
Mean vegetable, g per day^d^	225	193	255	177	249	192	172	219	203	194	186	194
Mean fibre intake, g per day^d^	19.4	16.1	20.1	16.1	20.3	16.4	16.7	17.6	16.0	17.6	16.3	17.3

CRC, colorectal cancer; d, day; NSAID, non-steroidal anti-inflammatory drug; wk, week.

a Socio-economic status is based on the Townsend deprivation index.^16^

b Family history is mother, father or sibling with colorectal cancer.

c Regular use of aspirin or ibuprofen.

d Mean fruit, vegetable, red-meat, processed-meat and fibre intakes are from the average of all 24-hour dietary assessments. The percentages of participants in the lowest and highest categories of intake from the baseline questionnaire who also completed the 24-hour dietary assessments were 42.8 and 38.5% for total red meat; 45.1 and 39.7% for processed meat; 46.3 and 41.6% for poultry; 38.2 and 41.0% for total fish; 42.7 and 39.2% for milk; and 36.9 and 50.4% for cheese.

**Table 2.** Association of lifestyle and anthropometric variables with intake of animal foods among women in UK Biobank

	Total red meat		Total processed meat		Poultry		Total fish		Dairy milk		Cheese	
	<1 time/wk	≥3 times/wk	Never	≥2 times/wk	Never	≥2 times/wk	<1 time/wk	≥3 times/wk	Never	≥300 mL/d	<1 time/wk	≥5 times/wk
*N*	32 006	49 425	32 232	52 706	15 803	124 377	19 803	73 995	8, 966	50 906	55 603	31 064
Mean (SD) age, years	54.5 (8.2)	56.8 (7.9)	55.7 (8.0)	55.7 (8.3)	54.1 (8.1)	55.5 (8.0)	53.5 (8.0)	57.3 (7.9)	55.6 (8.0)	57.1 (7.7)	56.6 (7.8)	55.3 (8.1)
% (*n*) highest fifth of socio-economic status^a^	16.3 (5215)	21.1 (10409)	17.3 (5566)	19.3 (10178)	15.8 (2487)	20.8 (25785)	14.6 (2893)	20.4 (15048)	17.0 (1519)	20.7 (10380)	18.2 (10077)	20.5 (10380)
% (*n*) current smokers	7.9 (2516)	10.1 (4971)	7.4 (2, 386)	10.8 (5669)	7.9 (1253)	8.3 (10 292)	13.5 (2666)	8.0 (5934)	13.0 (1167)	11.2 (5674)	9.4 (5235)	8.7 (2705)
Mean (SD) height, cm	162.5 (6.5)	162.2 (6.3)	162.3 (6.5)	162.4 (6.3)	162.8 (6.6)	162.4 (6.3)	162.0 (6.5)	162.4 (6.2)	162.3 (6.4)	162.5 (6.3)	161.6 (6.3)	163.7 (6.3)
Mean (SD) BMI, kg/m^2^	25.9 (5.0)	27.7 (5.3)	25.8 (4.9)	27.9 (5.6)	25.6 (4.9)	27.6 (5.4)	27.3 (5.6)	27.1 (5.2)	27.3 (5.9)	27.1 (5.0)	27.6 (5.3)	26.1 (5.1)
Mean (SD) waist circumference, cm	82.0 (12.3)	86.2 (12.7)	81.7 (12.0)	86.9 (13.3)	81.5 (12.2)	85.6 (12.7)	85.2 (13.4)	84.6 (12.5)	85.2 (13.9)	84.7 (12.3)	85.6 (12.7)	82.8 (12.5)
Mean (SD) body fat, %	34.8 (7.3)	37.5 (6.8)	34.7 (7.2)	37.6 (6.9)	34.3 (7.4)	37.2 (6.8)	36.6 (7.4)	36.5 (6.9)	36.5 (7.6)	36.7 (6.8)	37.3 (6.8)	34.9 (7.2)
% (*n*) family history of CRC^b^	8.3 (2653)	8.8 (4348)	8.7 (2797)	8.9 (4693)	8.1 (1281)	8.8 (10 931)	8.1 (1612)	9.0 (6664)	8.5 (761)	9.4 (4761)	8.7 (4862)	8.6 (2660)
% (*n*) regular NSAID use^c^	23.6 (7549)	26.7 (13 213)	23.0 (7414)	27.7 (14 574)	23.1 (3648)	26.7 (33 194)	26.4 (5233)	25.4 (18 761)	24.9 (2229)	26.1 (13 289)	26.2 (14 566)	24.4 (7580)
Mean (SD) alcohol, g per day	10.2 (11.0)	12.5 (12.4)	10.3 (11.0)	11.9 (12.4)	10.7 (11.4)	11.3 (11.4)	10.4 (12.2)	11.5 (11.2)	13.8 (13.7)	9.9 (10.2)	9.9 (10.8)	12.3 (12.2)
Mean (SD) no. of live births	2.2 (1.0)	2.3 (0.9)	2.2 (1.0)	2.3 (1.0)	2.2 (0.9)	2.3 (0.9)	2.3 (1.0)	2.2 (0.9)	2.2 (0.9)	2.3 (0.9)	2.3 (1.0)	2.2 (0.9)
% (*n*) post-menopausal	55.4 (17 737)	60.9 (30 102)	60.4 (19 475)	56.5 (29 757)	54.8 (8664)	56.6 (70 351)	50.0 (9901)	63.8 (47 171)	56.9 (5097)	63.3 (32 204)	61.1 (33 991)	57.6 (17 884)
% (*n*) ever HRT use	30.4 (9725)	40.3 (19 890)	34.1 (10 986)	36.4 (19 192)	27.9 (4402)	37.3 (46 404)	30.3 (5996)	41.0 (30 308)	37.0 (3315)	40.6 (20 640)	39.7 (22 090)	33.4 (10 374)
% (*n*) ever OCA use	78.7 (25 185)	80.3 (39 678)	77.8 (25 065)	80.9 (42 642)	79.2 (12 513)	82.7 (102 788)	80.6 (15 962)	79.0 (58 418)	82.5 (7392)	78.8 (40 129)	78.1 (43 421)	83.4 (25 890)
Mean red meat, g per day^d^	7	51	12	41	4	37	23	31	33	35	34	28
Mean processed meat, g per day^d^	7	20	5	25	2	19	13	15	17	17	17	15
Mean fruit, g per day^d^	219	191	232	180	216	200	187	221	210	212	215	198
Mean vegetable, g per day^d^	270	236	288	216	293	237	231	272	262	235	241	246
Mean fibre intake, g per day^c^	18.1	15.7	18.2	15.6	19.0	15.9	16.7	16.9	15.7	17.0	16.0	16.7

CRC, colorectal cancer; d, day; HRT, hormone replacement therapy; NSAID, non-steroidal anti-inflammatory drug; OCA, oral contraceptive use; wk, week.

a Socio-economic status is based on the Townsend deprivation index.^16^

b Family history is mother, father or sibling with colorectal cancer.

c Regular use of aspirin or ibuprofen.

d Mean fruit, vegetable, red-meat, processed-meat and fibre intakes are from the average of all 24-hour dietary assessments. The percentages of participants in the lowest and highest categories of intake from the baseline questionnaire who also completed the 24-hour dietary assessments were 45.2 and 39.8% for total red meat; 45.2 and 40.9% for processed meat; 50.2 and 41.8% for poultry; 40.2 and 41.8% for total fish; 46.7 and 39.3% for milk; and 38.0 and 52.0% for cheese.

The multivariable associations between foods, food groups and fibre and colorectal-cancer risk are shown in Figure 1 (red and processed meat, red meat, processed meat, poultry, fish, dairy milk and cheese) and Figure 2 (fruit, vegetables, fibre, alcohol, tea and coffee). The values from the multivariable model, as well as the results from the minimally adjusted model and the multivariable model in those that did not report a major change in their diet in the past 5 years because of illness are shown in Supplementary Tables 3 and 4, available as Supplementary data at *IJE* online. In the multivariable adjusted model, the HR for incident colorectal cancer was 1.20 [95% confidence interval (CI): 1.04–1.37] for those who reported consuming an average of 76 g/day of red and processed meat compared with those who reported consuming an average of 21 g/day [and per 50-g/day increment was 1.17 (95% CI: 1.04–1.32)]. For red meat, the HR was 1.15 (0.98–1.36) for those who reported consuming an average of 54 g/day compared with those who reported consuming an average of 8 g/day [and per 50-g/day increment was 1.18 (1.00–1.39)] and, for processed meat, the HR was 1.19 (1.01–1.41) for those who reported consuming an average of 29 g/day compared with those who reported consuming an average of 5 g/day [and per 25-g/day increment was 1.19 (1.03–1.38)]. We further adjusted the red and processed-meat analyses for milk, cheese and fibre from bread and breakfast cereals and the results were unchanged [the HR for each 50-g/day increment in red and processed meat was 1.18 (1.05–1.33)] for each 50-g/day increment in red meat was 1.19 (1.00–1.41) and for each 25-g/day increment in processed meat was 1.20 (1.03–1.40).

The HR for each 25-g/day increment in fish was 0.96 (0.86–1.07). The HR for each 100-mL/day increment in milk intake was 0.93 (0.87–1.01); we further adjusted the milk analyses for red meat, processed meat and fibre from bread and breakfast cereals and the HR for each 100-mL/day increment was 0.94 (0.87–1.02). The HR for each 5-g/day increment in total fibre was 0.94 (0.86–1.03) and for each 10-g/day increment in alcohol was 1.08 (1.04–1.12). We further adjusted the alcohol analyses for red meat, processed meat, milk, cheese and fibre from bread and breakfast cereals and the HR for each 10-g/day increment was 1.06 (1.03–1.10). The intakes of poultry, cheese, fruit, vegetables, tea and coffee were not associated with colorectal cancer. When we excluded participants who reported a major change in their diet in the past 5 years due to illness, the HRs were of similar magnitude.

When we adjusted for BMI instead of waist circumference, the results were very similar (data not shown). When we examined the effect of adjusting for each covariate sequentially (**Table 3**), using the total of red and processed meat as the main exposure, waist circumference attenuated the HR, as did alcohol and, to a lesser extent, smoking. Overall, the HR was attenuated by 7% after adjustment for 14 covariates. The HR (95% CI) for each 50-g/day increment in red-meat intake was 1.18 (1.00–1.39) and after adjustment for processed meat was 1.12 (0.94–1.34). The HR for each 50-g/day increment in processed-meat intake was 1.40 (1.04–1.88) and after adjustment for red meat was 1.33 (0.98–1.83).

**Table 3.** Hazard ratios (95% CIs) for the association with 50-g higher intake of red and processed meat per day and colorectal-cancer incidence with sequential adjustment for potential confounding variables

Level of adjustment	HR (95% CI)
Stratified by age, sex, deprivation and region	1.26 (1.12–1.42)
+ education	1.26 (1.12–1.41)
+ waist circumference	1.21 (1.08–1.36)
+ height	1.21 (1.08–1.36)
+ smoking	1.20 (1.07–1.35)
+ alcohol	1.17 (1.04–1.32)
+ physical activity	1.17 (1.04–1.32)
+ family history	1.17 (1.04–1.32)
+ NSAID use	1.17 (1.04–1.32)
+ vitamin D supplement use	1.17 (1.04–1.32)
+ folate supplement use	1.17 (1.04–1.32)
+ parity	1.17 (1.04–1.32)
+ menopause status	1.17 (1.04–1.32)
+ OCA use	1.17 (1.04–1.32)
+ HRT use	1.17 (1.04–1.32)

CI, confidence interval; HR, hazard ratio; HRT, hormone replacement therapy; NSAID, non-steroidal anti-inflammatory drug; OCA, oral contraceptive agent.

Supplementary Table 4, available as Supplementary data at *IJE* online, shows the main results in men and women separately. There was heterogeneity by sex for the associations between colorectal cancer and red and processed meat (*P*_heterogeneity_ = 0.008), with a positive association seen in men [HR for each 50-g/day increment in red-meat intake = 1.39 (1.17–1.64)] and no association was seen in women [HR for each 50-g/day increment in red meat = 0.99 (0.83–1.19)]. There was also heterogeneity by sex for red meat (*P*_heterogeneity_ = 0.008) and for processed meat (*P*_heterogeneity_ = 0.022). There was also heterogeneity by sex for alcohol, with a positive association seen in men [HR for each 10-g/day increment in alcohol intake = 1.12 (1.08–1.17)] and no association seen in women [HR for each 10-g/day increment = 0.99 (0.93–1.06), *P*_heterogeneity_ = 0.002]. There was no heterogeneity by sex for the associations between fish, dairy milk, cheese, fruit, vegetables, fibre, tea or coffee and colorectal cancer.

When we examined oily fish and non-oily fish separately, there were no associations between oily fish [HR for each 25-g/day increment = 0.87 (0.74–1.02)] or non-oily fish [HR for each 25-g/day increment = 0.94 (0.76–1.17)] and colorectal cancer (Supplementary Table 5, available as Supplementary data at *IJE* online).

When we examined the source of fibre, intake of fibre from bread and breakfast cereals was associated with a reduced risk of colorectal cancer [HR for the highest vs lowest fifth of intake = 0.86 (0.76–0.98), *P*_trend_ = 0.005]. We further adjusted the fibre from bread and breakfast cereals for red meat, processed meat, milk and cheese and the results were unchanged. Intakes of fruit and vegetable fibre were not associated with the risk of colorectal cancer (*P*_trend_ = 0.728 and *P*_trend_ = 0.633, respectively) (Supplementary Table 6, available as Supplementary data at *IJE* online).

When we examined the type of alcoholic drink, beer was associated with an increased risk of colorectal cancer [HR for each 10-g/day increment of alcohol from beer = 1.11 (1.06–1.18)]. The HR for each 10-g/day increment of alcohol from wine was 1.05 (1.00–1.10) and for each 10-g/day increment of alcohol from spirits was 1.08 (0.90–1.31) (Supplementary Table 7, available as Supplementary data at *IJE* online).

Supplementary Table 8, available as Supplementary data at *IJE* online, shows the results for colon and rectal cancer separately and Supplementary Table 9, available as Supplementary data at *IJE* online, shows the results for proximal and distal colon cancer separately. There was no heterogeneity between colon and rectal cancer for any of the foods or nutrients examined. There was heterogeneity between proximal and distal colon for red and processed meat, processed meat and alcohol; in all cases, a positive association was observed for distal colon cancer and no association for proximal colon cancer. When we examined the associations between type of alcoholic drink and colon and rectal cancer separately, the HR for each 10-g/day increment of alcohol from beer was 1.09 (1.02–1.16) for colon cancer and 1.16 (1.07–1.27) for rectal cancer, but there was no heterogeneity (*P*_heterogeneity_ = 0.223) (Supplementary Table 10, available as Supplementary data at *IJE* online).

## Discussion

In this large contemporary prospective study of half a million men and women from the UK that included 2609 cases of incident colorectal cancer, we found that a 25-g/day increment in processed-meat intake (equivalent to about one rasher of bacon or one slice of ham^21^) was associated with an 19% (95% CI: 3–38, *P*_trend_ = 0.020) greater risk of incident colorectal cancer. We also found that each 50-g/day increment in red-meat intake (equivalent to about one thick slice of roast beef or the edible portion of one lamb cutlet^21^) was associated with a 18% (95% CI: 0–39, *P*_trend_ = 0.049) greater risk of incident colorectal cancer. Each 10-g/day increment in alcohol intake [equivalent to about half a pint of 4.5% alcohol (by volume) beer] was associated with an 8% (95% CI: 4–12, *P*_trend_ < 0.001) greater risk of incident colorectal cancer. Intake of fibre from bread and breakfast cereals (but not fibre from vegetables or fruit) was also associated with a reduced risk.

The directions of our results for red and processed meat are in agreement with the latest systematic review from the WCRF Continuous Update Project, which summarized the evidence from available prospective studies for red and processed meat.^22^ However, the magnitudes of our estimates are nearly twice as large. This may be in part because we used re-measured intakes (using the data from the large sub-sample, *n* = 175 402) to fit the trends in risk per increment in mean intake. This approach reduces the impact of measurement error in the assessment of food intakes and measurement error tends to bias associations towards the null.^12^ Adjusting for waist circumference, alcohol and, to a lesser extent, smoking attenuated the results for red and processed meat. However, the fully adjusted models were adjusted for 18 variables that are known or suspected confounders and, for processed meat in particular, the fully adjusted results were still strong. We further adjusted red and processed meat for milk, cheese and fibre from bread and breakfast cereals and the results were unchanged. When we excluded participants who reported having made major changes to their diet in the past 5 years due to illness, the point estimates remained similar, indicating that reverse causality is not likely to explain the associations.

We found an increased risk for greater alcohol intake, in line with the WCRF review.^22^ When we looked at type of alcoholic drink, we found an association between beer and colorectal cancer. Our results for fibre are in agreement with those reported in a recent meta-analysis on fibre and colorectal cancer, which found that, in analyses by fibre type, only fibre from cereals, but not from fruit or vegetables, was inversely associated with risk.^23^ Different types of fibre may have different effects on stool transit time and weight, which may explain the different associations with colorectal cancer, but more research is needed to clarify the physiological effects of different types of fibre. Alternatively, the intake of fibre from cereals will be correlated with wholegrain intake, which has also been shown to be associated with a reduced risk of colorectal cancer.^23^ It is possible that phytochemicals or other non-fibre components of wholegrains are responsible for the observed associations. The WCRF review found an inverse association between fish and risk of colorectal cancer; however, this finding was driven by one study and was not robust to removal of that study from the meta-analysis.^22^ We did not find any associations between fruit and vegetables and colorectal cancer. The WCRF systematic review found a very weak inverse association between vegetables and colorectal cancer, which might be explained by residual confounding, and no association for fruit.^22^ In our study, the HR for 100-mL/day higher milk intake was 0.93 (95% CI: 0.87–1.01). The WCRF systematic review found an inverse association between milk intake and colorectal cancer [HR for 200 g/day = 0.94 (95% CI: 0.92–0.96)].^20^ The UK Biobank short food-frequency touchscreen questionnaire did not contain all sources of milk and therefore there may be more measurement error in our categorization of milk intakes, which would tend to bias the association towards the null.

Interestingly, we found heterogeneity by sex for red and processed meat, red meat, processed meat and alcohol, with the association stronger in men and null in women. The explanation for these differences by sex is unclear and could be a chance finding. However, there is evidence that the association between other factors, such as BMI^24^ and height^25^ and colorectal-cancer risk, differs between men and women. We also found heterogeneity by sub-site of the colon for the same food items—red and processed meat, processed meat and alcohol—with the associations stronger for distal cancer and null for proximal cancer, and previous work has shown that women are more likely to develop cancers in the proximal colon, whereas men are more likely to develop cancers in the distal colon.^26^ Future epidemiological studies examining dietary, lifestyle and anthropometric factors in relation to colorectal-cancer risk should examine associations by sex and by site and/or characteristics of the tumour.

Strengths of this study are the large number of participants and the ability to adjust for known and potential confounders. We have previously reported good reproducibility of the short food-frequency touchscreen questionnaire.^11^ This indicates that the participants’ reporting of food groups, including meat consumption, in UK Biobank is stable. We were also able to reduce the impact of regression dilution bias and other measurement errors, and quantify actual intakes, using the re-measured dietary intakes on the large sub-sample of participants who completed at least one 24-hour dietary assessment. Recruitment for UK Biobank is recent, from 2006 to 2010, so the results we present here indicate that red and processed meat, as is typically consumed in the present day, is associated with a higher risk of colorectal cancer. We were not able to adjust for total energy intake because the short food-frequency touchscreen questionnaire did not ask about every food group and therefore estimated total energy intake was not available. Importantly, we adjusted for measures of body size (waist circumference in the main analysis and BMI in a sensitivity analysis) and physical activity; adjusting for body size and physical activity has been shown to better approximate objectively measured total energy expenditure (and therefore true energy intake) than estimated energy intake from a food-frequency questionnaire.^27^ A large sub-sample completed a 24-hour dietary assessment during follow-up. Previous work has shown that UK Biobank participants who completed at least one 24-hour dietary assessment were more likely to be women, older, of white ethnicity, less deprived and more educated than those that did not complete a 24-hour dietary assessment.^14^ Nevertheless, the approach we used to calculate the trends based on re-measured intakes has been shown to be robust to various factors influencing which participants provide a second measure.^28^ We did not have information about participation in bowel-screening programmes during follow-up and participants who underwent bowel screening would be more likely to be diagnosed with bowel cancer and it is possible that participating in screening could be associated with dietary intakes. Although this study is one of the largest single studies to report on dietary factors and cancers at sub-sites of the colorectum, more data are needed for reliable estimation of the associations between dietary factors and sub-sites of the colorectum.

## Conclusions

In conclusion, in this systematic analysis of a contemporary cohort of half a million men and women from the UK population, we found that consumption of red and processed meat and alcohol was associated with an increased risk of colorectal cancer. We also found that fibre from bread and breakfast cereals was associated with a reduced risk. The current recommendation by the UK Government Department of Health is that people should not eat more than 90 g of red and processed meat a day.^29^ Participants in the highest category of red and processed-meat consumption were consuming an average of 76 g of red and processed meat per day and thus this group was on average meeting the current recommendation but still had a 20% (95% CI: 4–37) increase in risk of colorectal cancer compared with those who ate an average of 21 g of red and processed meat per day. Therefore, our results suggest that reductions in meat intake below the current recommendation may further reduce the risk of colorectal cancer.

## Funding

This work was supported by Girdlers’ New Zealand Health Research Council Fellowship (K.E.B.), Cancer Research UK (T.J.K.; C8221/A19170 and 570/A16491), the UK Medical Research Council (T.J.K.; MR/M012190/1) and the Wellcome Trust [T.J.K.; Our Planet Our Health, project: Livestock, Environment and People (LEAP) (205212/Z/16/Z)]. The funders did not influence the design of the study, analysis or interpretation of the data, the writing of this report or the decision to publish.

## Supplementary Material

dyz064_Supplementary_Data
