# Supplementary material for: Diet and colorectal cancer in UK Biobank: a prospective study
Source: Int J Epidemiol. 2019 Apr 17;49(1):246–58. doi: 10.1093/ije/dyz064 (PMC7124508; doi:10.1093/ije/dyz064)
Supplement: dyz064_Supplementary_Data [file dyz064_supplementary_data.zip › dyz064-suppl_data/ije-2018-05-0670-File005.docx]

**Supplementary methods**

**Exposure variables**

**Dietary variables**

Generally, we grouped participants into four categories according to the intake of each food/food group. The cut-points for the categories were whole integers, chosen to have reasonable and similar numbers of participants in each group, as far as the distribution of data allowed.

**Meat and fish**

For oily fish, non-oily fish, processed meats, poultry, beef, lamb, pork, participants were asked how often each item was consumed with possible answers being: ‘never, ‘less than once a week’, ‘once a week’, ‘2-4 times a week’, ‘5-6 times a week’, ‘once or more daily’, ‘do not know’, ‘prefer not to answer’. For processed meat, poultry, oily fish and non-oily fish, we combined the top three frequencies to get four categories: never, <1.0 time per week, 1.0 time per week, and ≥2.0 times per week’. To rank the participants by weekly red meat consumption based on the touchscreen, we summed the frequencies for beef, pork, and lamb/mutton, using the following coding: ‘Never’ = 0, ‘Less than once a week’ = 0.5, ‘Once a week’ = 1, ‘2-4 times a week’ = 3, ‘5-6 times a week’ = 5.5, ‘Once or more daily’ = 7. The four categories for red meat consumption were: <1 time per week, 1.0-1.9 times per week, 2.0-2.9 times per week, and ≥3.0 times per week. For red and processed meat, we summed the frequencies for beef, pork, lamb/mutton, and processed meat. The categories for red and processed meat consumption were: < 2.0 times per week, 2.0-2.9 times per week, 3.0-3.9 times per week, and ≥4.0 times per week. To rank the participants by weekly total fish consumption based on the touchscreen, we summed the frequencies for oily fish and non-oily fish and grouped participants into the following categories: <1.0 time per week, 1.0-1.9 times per week, 2.0-2.9 times per week, and ≥3.0 times per week.

**Fruit**

For fruit, participants were asked to direct enter the number of pieces of fresh fruit and dried fruit (with examples given as to what constitutes a piece eaten per day) or select ‘less than one’, ‘do not know’ or ‘prefer not to answer’. One piece of fresh fruit, and two ‘pieces’ of dried fruit were counted as a serving. We grouped participants into the following categories: <2.0 servings per day, 2.0-2.9 servings per day, 3.0-3.9 servings per day, and ≥4.0 servings per day.

**Vegetables**

For vegetables, participants were asked to direct enter the number of heaped tablespoons of cooked vegetables and salad/raw vegetables eaten per day or select ‘less than one’, ‘do not know’ or ‘prefer not to answer’. Two heaped tablespoons of vegetables were counted as a serving. We grouped participants into the following categories: <2.0 servings per day, 2.0-2.9 servings per day, 3.0-3.9 servings per day, and ≥4.0 servings per day.

**Milk**

We derived an estimate of milk intake using the questions on type of milk, bowls of breakfast cereal, cups of tea, and cups of coffee. Participants were asked which type of milk they mainly use, and those who answered ‘Never/rarely have milk’ were assigned to the first category. For the participants who selected one of ‘Full cream’, ‘Semi-skimmed’ or ‘Skimmed’, we estimated their total milk consumption by summing up their daily consumption of milk assuming that participants added 100 mL of milk to each bowl of breakfast cereal, 35 mL of milk to each cup of tea, and 25 mL of milk to each cup of coffee. These participants were then divided into three categories: those that consumed <150 mL of milk, 150-299 mL of milk, and ≥300 mL of milk daily. A limitation of the estimated milk intake is that some people may not add milk to tea or coffee, but the touchscreen did not ask about this. To determine whether this derived estimate of milk intake was able to discriminate between participants with low and high milk intakes, we used the sub-sample of participants who completed at least one 24-hour dietary assessment. Within each category of milk consumption from the touchscreen questionnaire, we calculated the average intake of dairy milk from the 24-hour dietary assessments. This showed that the touchscreen categories of milk consumption were able to discriminate between those with low and high intakes (see Supplementary Table 1). We further calculated that within the 24-hour dietary assessments, for those that selected whole milk, semi-skimmed milk, or skim milk as the milk mainly used, 94 % of their total milk intake came from milk added to breakfast cereal, tea, and coffee (the remainder was from glasses of milk and milky drinks, e.g. hot chocolate, that were not asked about in the touchscreen questionnaire).

**Supplementary Table 1**

Estimated dairy milk intake from the touchscreen questionnaire in relation to the 24-hour dietary assessments

| Estimated milk intake from the touchscreen | Mean milk intake from the 24-hour dietary assessments |
| --- | --- |
| \| Never have milk \| \| --- \| | 27 mL |
| < 150 mL dairy milk | 151 mL |
| 150-299 mL dairy milk | 224 mL |
| ≥ 300 mL dairy milk | 283 mL |

**Cheese**

For cheese, participants were asked how often each item was consumed with possible answers being: ‘never, ‘less than once a week’, ‘once a week’, ‘2-4 times a week’, ‘5-6 times a week’, ‘once or more daily’, ‘do not know’, ‘prefer not to answer’. We combined the bottom two frequencies and the top two frequencies to get four categories: <1.0 time per week, 1.0 time per week, 2.0-4.9 times per week, and ≥5.0 times per week.

**Tea and coffee**

For amount of tea and coffee, participants were asked to direct enter how many cups of tea, including black and green tea, and coffee (including decaffeinated coffee they drank each day, or select ‘less than one’, ‘do not know’, or ‘prefer not to answer’. For tea, we grouped participants into the following categories: <2.0 cups per day, 2.0-3.9 cups per day, 4.0-5.9 cups per day, and ≥6.0 cups per day. For coffee, we grouped participants into the following categories: 0 cups/day, 0.5-1.9 cups per day, 2.0-2.9 cups per day, and ≥3.0 cups per day.

**Major changes in diet**

For changes in diet, participants were asked if they made any major changes to their diet in the past 5 years with possible answers being ‘no’, ‘yes, because of illness’, ‘yes, because of other reasons’, ‘prefer not to answer’.

**Covariates**

**Education**

For education, participants were asked ‘Which of the following qualification do you have? (You can select more than one). Possible answers were: College or University degree; A levels/AS levels or equivalent; O levels/GCSEs or equivalent; CSEs or equivalent; NVQ or HND or HNC or equivalent; Other professional qualifications eg: nursing, teaching; None of the above; Prefer not to answer. We grouped participants into the following categories, based on their highest reported level of education: (College or University degree, vocational qualifications (other professional qualifications/NVQ or HND or HNC), optional national exams at ages 17 to 18 years (A levels/AS levels), national exams at age 16 years (O levels/GCSEs/CSEs), none of the above, unknown (prefer not to answer)).

**Region**

Analyses were stratified by region of recruitment centre, we grouped assessment centres into 10 regions corresponding approximately to the areas covered by the cancer registries: London (assessment centres: St Bartholomew’s Hospital, Hounslow, Croydon) Wales (assessment centres: Swansea, Wrexham, Cardiff), North-West England (assessment centres: Stockport, Manchester, Liverpool, Bury), North-East England (assessment centres: Newcastle, Middlesbrough), Yorkshire (assessment centres: Leeds, Sheffield), West Midlands (assessment centres: Stoke, Birmingham) East Midlands (assessment centre: Nottingham), South-East England (assessment centres: Oxford, Reading), South-West England (assessment centre: Bristol), Scotland (assessment centres: Glasgow, Edinburgh)).

**Alcohol**

Participants were asked how often they drank alcohol with the possible answers being: “daily or almost daily”, “three or four times a week”, “once or twice a week”, “one to three times a month”, “special occasions only”, “never”, “prefer not to answer”. Participants were also asked separately about their weekly and monthly consumption of pints of beer, glasses of red wine, glasses of white wine/champagne, glasses of fortified wine, measures of spirits/liqueurs and glasses of other alcohol. We assumed a pint of beer contained 20 g of alcohol, and all other drinks contained 10 g, and summed their total weekly and monthly consumption of alcohol accordingly. If the participant reported ‘do not know’ or ‘prefer not to answer’ to one of these questions on weekly or monthly consumption, they were coded as missing, except for ‘other alcohol’, in which case we assigned them 0 g from other alcohol. We preferentially used participants’ reported weekly consumption of alcohol, if this was unknown (due to the participant reporting ‘do not know’ or ‘prefer not to answer’ for one or more of the relevant questions, except for ‘other alcohol’) we used monthly consumption, if available. To get an estimated daily total, we divided weekly consumption by 7 (or monthly consumption by 30.4375). We categorised alcohol consumption as <1 g/day, 1-7 g/day, 8-15 g/day, and ≥16 g/day, or unknown. We calculated the median intakes of alcohol in grams per day for participants in each of the following categories of overall alcohol intake frequency: “daily or almost daily”, “three or four times a week”, “once or twice a week”, and “one to three times per month”. For participants who had unknown grams/day of alcohol but who reported one of these top four categories of overall alcohol intake frequency, we assigned them the median value (g/day) from their category. For participants who had unknown grams/day of alcohol but who reported an overall alcohol intake frequency of ‘never’ or ‘special occasions’, we assigned them to the category of ‘<1 g/day’.
