# Supplementary material for: Diet and colorectal cancer in UK Biobank: a prospective study
Source: Int J Epidemiol. 2019 Apr 17;49(1):246–58. doi: 10.1093/ije/dyz064 (PMC7124508; doi:10.1093/ije/dyz064)
Supplement: dyz064_Supplementary_Data [file dyz064_supplementary_data.zip › dyz064-suppl_data/ije-2018-05-0670-File006.docx]

| **Supplementary Table 1** Association of lifestyle and anthropometric variables with intake of plant foods, fibre, and alcohol among men in UK Biobank | | | | | | | | | | | | | | | | |
| --- | --- | --- | --- | --- | --- | --- | --- | --- | --- | --- | --- | --- | --- | --- | --- | --- |
|  | Total fruit | | | Total vegetables | | | | Total fibre | | | Total alcohol | | Tea | | Coffee | |
|  | <2 ser/day | ≥4 ser/day | | ≤ 2 ser/day | | ≥ 4 ser/day | | Lowest fifth | | Highest fifth | <1g/day | ≥ 16 g/day | <2 cups/day | ≥6 cups/day | 0 cups/day | ≥3 cups/day |
| n | 86,409 | 39,751 | | 84,895 | | 26,847 | | 49,128 | | 40,928 | 28,819 | 108,266 | 57,362 | 42,109 | 44,430 | 76,183 |
| Mean (SD) age, years | 55.9 (8.3) | 57.4 (8.0) | | 55.6 (8.2) | | 57.1 (8.2) | | 55.1 (8.3) | | 57.4 (8.0) | 56.4 (8.5) | 56.6 (8.0) | 55.3 (8.4) | 57.0 (7.9) | 55.9 (8.3) | 56.2 (8.1) |
| % (n) highest fifth of socioeconomic status* | 19.3 (16639) | 19.6 (7781) | | 19.8 (16811) | | 18.3 (4900) | | 17.9 (8782) | | 20.6 (8432) | 13.1 (3774) | 20.4 (22102) | 19.1 (10946) | 19.0 (7975) | 16.5 (7311) | 19.0 (7975) |
| % (n) current smokers | 26.3 (7135) | 8.2 (5370) | | 14.4 (12 237) | | 11.7 (3151) | | 21.0 (10 343) | | 7.6 (3099) | 14.6 (4198) | 15.0 (16 288) | 14.3 (8226) | 16.4 (6896) | 12.2 (5405) | 16.3 (12 381) |
| Mean (SD) height, cm | 175.5 (6.9) | 175.7 (6.9) | | 175.6 (6.8) | | 175.4 (6.9) | | 175.0 (6.9) | | 176.2 (6.9) | 174.2 (7.0) | 175.8 (6.7) | 175.8 (6.9) | 175.4 (6.8) | 174.8 (7.0) | 176.1 (6.8) |
| Mean (SD) BMI, kg/m^2^ | 27.8 (4.2) | 28.0 (4.4) | | 27.8 (4.3) | | 28.0(4.3) | | 28.1 (4.3) | | 27.6 (4.3) | 28.2 (5.0) | 28.0 (4.0) | 28.2 (4.5) | 27.9 (4.3) | 27.9 (4.4) | 28.1 (4.3) |
| Mean (SD) waist circumference, cm | 97.2 (11.2) | 96.9 (11.7) | | 96.8 (11.4) | | 97.2 (11.5) | | 97.7 (11.4) | | 96.0 (11.6) | 97.9 (13.2) | 97.3 (10.7) | 97.7 (11.9) | 97.1 (11.5) | 96.9 (11.7) | 97.7 (11.4) |
| Mean (SD) body fat, % | 25.5 (5.7) | 25.1 (6.0) | | 25.3 (5.8) | | 25.3 (5.9) | | 25.9 (5.7) | | 24.4 (6.0) | 25.7 (6.4) | 25.5 (5.5) | 25.6 (5.9) | 25.2 (5.9) | 25.5 (5.9) | 25.4 (5.8) |
| % (n) family history of CRC^†^ | 8.2 (2213) | 8.9 (5892) | | 8.5 (7245) | | 8.8 (2349) | | 8.4 (4106) | | 8.9 (3658) | 7.6 (2201) | 9.1 (9888) | 8.1 (4660) | 9.2 (3863) | 8.4 (3711) | 8.7 (6595) |
| % (n) regular NSAID use^‡^ | 28.3 (7685) | 29.5 (19 463) | | 27.4 (23 248) | | 29.8 (8001) | | 28.1 (13 788) | | 28.9 (11 833) | 31.3 (9031) | 28.5 (30 853) | 27.8 (15 945) | 30.0 (12 637) | 27.9 (12 407) | 28.9 (22 030) |
| Mean (SD) alcohol, g per day^§^ | 27.9 (26.7) | 20.6 (19.8) | | 24.5 (24.5) | | 24.7 (23.9) | | 29.5 (28.6) | | 20.9 (20.0) | 0.4 (0.4) | 37.7 (23.4) | 25.8 (25.2) | 23.8 (23.9) | 25.2 (26.7) | 24.7 (22.5) |
| Mean red meat, g per day^§^ | 47 | 37 | | 44 | | 39 | | 50 | | 36 | 38 | 46 | 43 | 44 | 43 | 44 |
| Mean processed meat, g per day^§^ | 28 | 21 | | 27 | | 22 | | 30 | | 20 | 22 | 26 | 25 | 26 | 25 | 26 |
| Mean fruit, g per day^§^ | 94 | 309 | | 151 | | 227 | | 91 | | 275 | 205 | 164 | 170 | 182 | 180 | 173 |
| Mean vegetable, g per day^§^ | 164 | 230 | | 138 | | 272 | | 133 | | 251 | 181 | 194 | 176 | 194 | 186 | 184 |
| Mean fibre intake, g per day^§^ | 14.4 | 19.6 | | 15.3 | | 18.6 | | 12.8 | | 20.5 | 17.3 | 16.0 | 15.8 | 17.2 | 16.6 | 16.2 |
| CRC: Colorectal cancer; d: day; NSAID: non-steroidal, anti-inflammatory drug; wk: week | | | | | | | | | | | | | |  |  |  |
| *Socioeconomic status is based on the Townsend deprivation index (16) | | | | | | |  | |  |  |  |  |  |  |  |  |
| ^†^Family history is mother, father, or sibling with colorectal cancer | | | | | | |  | |  |  |  |  |  |  |  |  |
| ^‡^Regular use of aspirin or ibuprofen | |  |  | |  | |  | |  |  |  |  |  |  |  |  |
| ^§^Mean fruit, vegetable, red meat, processed meat, and fibre intakes are from the average of all 24-hour dietary assessments | | | | | | | | | | |  |  |  |  |  |  |

| **Supplementary Table 2** Association of lifestyle and anthropometric variables with plant foods, fibre, and alcohol among women in UK Biobank | | | | | | | | | | | | | | | | | | | | | |
| --- | --- | --- | --- | --- | --- | --- | --- | --- | --- | --- | --- | --- | --- | --- | --- | --- | --- | --- | --- | --- | --- |
|  | Total fruit | | | | Total vegetables | | | | Total fibre | | | | Total alcohol | | | | Tea | | | Coffee | |
|  | <2 ser/day | ≥4 ser/day | | | ≤ 2 ser/day | | ≥ 4 ser/day | | Lowest fifth | | Highest fifth | | <1g/day | | ≥ 16 g/day | | <2 cups/day | | ≥6 cups/day | 0 cups/day | ≥3 cups/day |
| n | 68,269 | 64,215 | | | 76,411 | | 37,354 | | 42,085 | | 49,360 | | 63,949 | | 48,262 | | 67,348 | | 47,151 | 60,844 | 74,415 |
| Mean (SD) age, years | 54.5 (8.2) | 57.7 (7.5) | | | 55.3 (8.2) | | 56.3 (7.8) | | 53.9 (8.2) | | 57.6 (7.6) | | 57.0 (8.1) | | 55.4 (7.8) | | 55.3 (8.2) | | 56.6 (7.7) | 54.8 (8.1) | 56.5 (7.9) |
| % (n) highest fifth of socioeconomic status* | 18.1 (12330) | 20.5 (13143) | | | 19.2 (14645) | | 18.4 (6875) | | 16.8 (7057) | | 20.5 (10082) | | 15.1 (9639) | | 20.2 (9722) | | 18.4 (12388) | | 19.5 (9159) | 17.5 (10629) | 20.5 (15266) |
| % (n) current smokers | 15.2 (10 367) | 5.3 (3421) | | | 11.0 (8392) | | 8.3 (3114) | | 18.0 (7582) | | 5.4 (2664) | | 9.7 (6173) | | 14.3 (6876) | | 11.0 (7416) | | 11.7 (5536) | 8.1 (4941) | 13.2 (9813) |
| Mean (SD) height, cm | 162.4 (6.4) | 162.5 (6.3) | | | 162.3 (6.3) | | 162.5 (6.4) | | 162.0 (6.4) | | 162.7 (6.3) | | 161.3 (6.4) | | 163.2 (6.2) | | 162.6 (6.3) | | 162.3 (6.3) | 162.0 (6.4) | 162.6 (6.2) |
| Mean (SD) BMI, kg/m^2^ | 27.2 (5.3) | 26.9 (5.1) | | | 27.1 (5.3) | | 27.2 (5.2) | | 27.4 (5.4) | | 26.9 (5.2) | | 28.4 (6.0) | | 26.6 (4.6) | | 27.5 (5.6) | | 27.2 (5.1) | 27.3 (5.4) | 27.5 (5.2) |
| Mean (SD) waist circumference, cm | 85.2 (12.7) | 84.2 (12.5) | | | 84.7 (12.8) | | 84.7 (12.6) | | 85.7 (12.9) | | 84.0 (12.6) | | 87.5 (14.1) | | 84.1 (11.6) | | 85.6 (13.4) | | 84.6 (12.4) | 85.1 (13.0) | 85.5 (12.7) |
| Mean (SD) body fat % | 36.8 (7.0) | 36.2 (7.0) | | | 36.6 (7.0) | | 36.4 (7.0) | | 37.1 (7.0) | | 36.0 (7.1) | | 37.9 (7.3) | | 36.1 (6.6) | | 36.9 (7.2) | | 36.8 (6.8) | 36.8 (7.0) | 37.0 (6.9) |
| % (n) family history of CRC^†^ | 8.5 (5,807) | 9.2 (5905) | | | 8.7 (6621) | | 9.0 (3352) | | 8.1 (3417) | | 9.5 (4690) | | 8.6 (5490) | | 9.0 (4354) | | 8.4 (5636) | | 9.3 (4406) | 8.7 (5313) | 9.0 (6684) |
| % (n) regular NSAID use^‡^ | 26.7 (18 200) | 24.9 (15 979) | | | 25.5 (19 448) | | 25.5 (9541) | | 27.5 (11 577) | | 24.9 (12 309) | | 26.2 (16 732) | | 26.5 (12 787) | | 25.4 (17 096) | | 26.2 (12 374) | 24.9 (15 166) | 26.5 (19 730) |
| Mean (SD) alcohol, g per day | 13.0 (13.5) | 9.7 (9.5) | | | 10.6 (11.4) | | 11.8 (11.8) | | 12.8 (14.1) | | 10.1 (9.9) | | 0.4 (0.4) | | 26.6 (12.5) | | 12.0 (12.4) | | 10.4 (10.9) | 10.3 (12.1) | 11.9 (11.4) |
| Mean (SD) no. of live births | 2.2 (0.9) | 2.3 (0.9) | | | 2.2 (0.9) | | 2.2 (0.9) | | 2.2 (1.0) | | 2.3 (0.9) | | 2.4 (1.1) | | 2.2 (0.9) | | 2.2 (0.9) | | 2.3 (0.9) | 2.3 (1.0) | 2.2 (0.9) |
| % (n) post-menopausal | 52.7 (35 997) | 66.5 (42 691) | | | 56.5 (43 158) | | 60.7 (22 683) | | 50.2 (21 111) | | 66.0 (32 549) | | 61.4 (39 236) | | 58.0 (27 989) | | 56.1 (37 744) | | 61.5 (29 007) | 54.3 (33 013) | 61.0 (45 411) |
| % (n) ever HRT use | 33.8 (23 092) | 41.6 (26 684) | | | 35.4 (27 017) | | 38.1 (14 230) | | 32.8 (13 781) | | 40.8 (20 149) | | 61.3 (39 202) | | 61.5 (29 694) | | 35.0 (23 549) | | 39.9 (18 802) | 34.8 (21 195) | 38.4 (28 584) |
| % (n) ever OCA use | 83.0 (56 626) | 78.4 (50 351) | | | 81.6 (62 320) | | 79.5 (29 699) | | 83.4 (35 114) | | 78.3 (38 632) | | 71.2 (45 512) | | 87.7 (42 326) | | 82.5 (55 555) | | 79.9 (37 675) | 80.2 (48 763) | 82.3 (61 230) |
| Mean red meat, g per day^§^ | 37 | 30 | | | 35 | | 30 | | 38 | | 29 | | 31 | | 37 | | 33 | | 35 | 33 | 34 |
| Mean processed meat, g per day^§^ | 19 | 14 | | | 18 | | 15 | | 20 | | 14 | | 16 | | 18 | | 17 | | 17 | 17 | 18 |
| Mean fruit, g per day^§^ | 102 | 302 | | | 165 | | 246 | | 103 | | 293 | | 220 | | 172 | | 195 | | 204 | 196 | 201 |
| Mean vegetable, g per day^§^ | 197 | 278 | | | 163 | | 330 | | 160 | | 306 | | 230 | | 244 | | 233 | | 238 | 230 | 234 |
| Mean fibre intake, g per day^‡^ | 13.7 | 18.5 | | | 14.6 | | 17.9 | | 12.4 | | 19.4 | | 16.7 | | 15.1 | | 15.5 | | 16.6 | 16.0 | 15.9 |
| CRC: colorectal cancer; d: day; HRT: hormone replacement therapy; NSAID: non-steroidal, anti-inflammatory drug; OCA: oral contraceptive use; wk: week | | | | | | | | | | | | | | | | | | |  |  |  |
| *Socioeconomic status is based on the Townsend deprivation index (16) | | | | | | | |  | |  | |  | |  | |  | |  |  |  |  |
| ^†^Family history is mother, father, or sibling with colorectal cancer | | | | | | | |  | |  | |  | |  | |  | |  |  |  |  |
| ^‡^Regular use of aspirin or ibuprofen | | |  |  | |  | |  | |  | |  | |  | |  | |  |  |  |  |
| ^§^Mean fruit, vegetable, red meat, processed meat, and fibre intakes are from the average of all 24-hour dietary assessments | | | | | | | | | | | | | |  | |  | |  |  |  |  |

| **Supplementary Table 3** Associations between dietary factors and colorectal cancer in UK Biobank | | | | | | | |  | |  | |  | |
| --- | --- | --- | --- | --- | --- | --- | --- | --- | --- | --- | --- | --- | --- |
|  | **All participants (n=475 581)** | | | | |  | **Participants who did not report changing their diet due to illness (n=425,112)^*^** | | | | | | |
| Reported consumption at recruitment | Mean intake (g/day)^†^ | Participants | Cases | HR (95% CI) from minimally adjusted model^‡^ | HR (95% CI) from fully adjusted model^§^ |  | Mean intake (g/day)^†^ | | Participants | | Cases | | HR (95% CI) from fully adjusted model^§^ |
| Red and processed meat |  |  |  |  |  |  |  | |  | |  | |  |
| <2.0 times/week | 21 | 68 359 | 274 | 1.00 (Ref) | 1.00 (Ref) |  | 21 | | 60 503 | | 236 | | 1.00 (Ref) |
| 2.0-2.9 times/week | 52 | 135 973 | 704 | 1.15 (1.00-1.32) | 1.10 (0.96-1.27) |  | 52 | | 122 763 | | 623 | | 1.12 (0.96-1.30) |
| 3.0-3.9 times/week | 64 | 71 391 | 388 | 1.15 (0.98-1.34) | 1.09 (0.93-1.27) |  | 64 | | 64 119 | | 341 | | 1.10 (0.93-1.30) |
| ≥4.0 times/week | 76 | 192 600 | 1209 | 1.29 (1.13-1.48) | 1.20 (1.04-1.37) |  | 75 | | 171 817 | | 1045 | | 1.20 (1.04-1.40) |
| per 50 g/day |  |  |  | 1.30 (1.10-1.53) | 1.17 (1.04-1.32) |  |  | |  | |  | | 1.18 (1.04-1.34) |
|  |  |  |  | Ptrend=0.002 | Ptrend=0.008 |  |  | |  | |  | | Ptrend=0.012 |
| Red meat |  |  |  |  |  |  |  | |  | |  | |  |
| <1.0 time/week | 8 | 47 795 | 187 | 1.00 (Ref) | 1.00 (Ref) |  | 7 | | 42 470 | | 161 | | 1.00 (Ref) |
| 1.0-1.9 times/week | 34 | 184 816 | 947 | 1.14 (0.97-1.34) | 1.09 (0.93-1.28) |  | 34 | | 165 861 | | 838 | | 1.11 (0.94-1.32) |
| 2.0-2.9 times/week | 44 | 131 486 | 781 | 1.24 (1.06-1.46) | 1.16 (0.99-1.37) |  | 44 | | 118 422 | | 682 | | 1.17 (0.98-1.39) |
| ≥ 3.0 times/week | 54 | 104 813 | 661 | 1.25 (1.06-1.48) | 1.15 (0.98-1.36) |  | 54 | | 92 901 | | 565 | | 1.16 (0.97-1.39) |
| per 50 g/day |  |  |  | 1.26 (1.12-1.42) | 1.18 (1.00-1.39) |  |  | |  | |  | | 1.17 (0.98-1.40) |
|  |  |  |  | Ptrend<0.001 | Ptrend=0.049 |  |  | |  | |  | | Ptrend=0.078 |
| Processed meat |  |  |  |  |  |  |  | |  | |  | |  |
| Never | 5 | 44 107 | 175 | 1.00 (Ref) | 1.00 (Ref) |  | 5 | | 38 870 | | 153 | | 1.00 (Ref) |
| <1.0 time/week | 16 | 143 673 | 728 | 1.15 (0.97-1.35) | 1.09 (0.92-1.29) |  | 16 | | 129 225 | | 637 | | 1.07 (0.90-1.28) |
| 1.0 time/week | 22 | 138 239 | 781 | 1.21 (1.03-1.43) | 1.14 (0.96-1.34) |  | 22 | | 123 992 | | 678 | | 1.11 (0.93-1.33) |
| ≥2.0 times/week | 29 | 147 417 | 913 | 1.30 (1.10-1.53) | 1.19 (1.01-1.41) |  | 29 | | 131 288 | | 797 | | 1.19 (0.99-1.42) |
| per 25 g/day |  |  |  | 1.30 (1.12-1.50) | 1.19 (1.03-1.38) |  |  | |  | |  | | 1.20 (1.02-1.41) |
|  |  |  |  | Ptrend<0.001 | Ptrend=0.020 |  |  | |  | |  | | Ptrend=0.024 |
| Poultry |  |  |  |  |  |  |  | |  | |  | |  |
| Never | 2 | 24 328 | 108 | 1.00 (Ref) | 1.00 (Ref) |  | 2 | | 21 910 | | 90 | | 1.00 (Ref) |
| <1.0 time/week | 19 | 50 801 | 283 | 0.93 (0.74-1.16) | 0.89 (0.71-1.11) |  | 19 | | 43 548 | | 243 | | 0.91 (0.72-1.17) |
| 1.0 time/week | 28 | 169 686 | 1016 | 1.04 (0.85-1.27) | 0.99 (0.81-1.21) |  | 28 | | 152 606 | | 896 | | 1.05 (0.84-1.31) |
| ≥2.0 times/week | 40 | 228 784 | 1188 | 1.03 (0.84-1.25) | 0.96 (0.79-1.18) |  | 40 | | 203 409 | | 1033 | | 1.02 (0.82-1.27) |
| per 25 g/day |  |  |  | 1.04 (0.94-1.15) | 1.01 (0.91-1.12) |  |  | |  | |  | | 1.04 (0.93-1.16) |
|  |  |  |  | Ptrend=0.409 | Ptrend=0.851 |  |  | |  | |  | | Ptrend=0.461 |
| Total fish |  |  |  |  |  |  |  | |  | |  | |  |
| <1.0 time/week | 6 | 36 512 | 165 | 1.00 (Ref) | 1.00 (Ref) |  | 6 | | 32 361 | | 147 | | 1.00 (Ref) |
| 1.0-1.9 times/week | 21 | 188 934 | 1,007 | 1.01 (0.85-1.19) | 0.98 (0.83-1.16) |  | 21 | | 171 349 | | 873 | | 0.95 (0.79-1.13) |
| 2.0-2.9 times/week | 29 | 111 375 | 643 | 1.00 (0.84-1.18) | 0.98 (0.82-1.16) |  | 29 | | 100 224 | | 568 | | 0.96 (0.80-1.16) |
| ≥3.0 times/week | 39 | 133 270 | 761 | 0.97 (0.82-1.15) | 0.95 (0.80-1.13) |  | 39 | | 116 615 | | 660 | | 0.95 (0.79-1.14) |
| per 25 g/day |  |  |  | 0.97 (0.87-1.07) | 0.96 (0.86-1.07) |  |  | |  | |  | | 0.99 (0.88-1.11) |
|  |  |  |  | Ptrend=0.541 | Ptrend=0.470 |  |  | |  | |  | | Ptrend=0.820 |
| Dairy milk |  |  |  |  |  |  |  | |  | |  | |  |
| never | 30 | 15 823 | 85 | 1.00 (Ref) | 1.00 (Ref) |  | 29 | | 13 757 | | 70 | | 1.00 (Ref) |
| <150 mL/day | 157 | 84 707 | 466 | 1.11 (0.88-1.40) | 1.10 (0.87-1.38) |  | 157 | | 75 694 | | 409 | | 1.15 (0.89-1.48) |
| 150-299 mL/day | 223 | 249 393 | 1,404 | 0.97 (0.78-1.21) | 0.99 (0.79-1.23) |  | 223 | | 225 574 | | 1,240 | | 1.03 (0.81-1.31) |
| ≥300 mL/day | 279 | 99 809 | 540 | 0.91 (0.72-1.14) | 0.94 (0.74-1.18) |  | 280 | | 88 650 | | 464 | | 0.97 (0.75-1.25) |
| per 100 mL/day |  |  |  | 0.91 (0.85-0.98) | 0.93 (0.87-1.01) |  |  | |  | |  | | 0.94 (0.86-1.01) |
|  |  |  |  | Ptrend=0.016 | Ptrend=0.070 |  |  | |  | |  | | Ptrend=0.110 |
| Cheese |  |  |  |  |  |  |  | |  | |  | |  |
| <1.0 time/week | 13 | 92 894 | 498 | 1.00 (ref) | 1.00 (ref) |  | 14 | | 79 627 | | 418 | | 1.00 (ref) |
| 1.0 time/week | 18 | 99 265 | 541 | 1.00 (0.88-1.13) | 0.98 (0.87-1.11) |  | 18 | | 88 164 | | 467 | | 0.97 (0.85-1.11) |
| 2.0-4.9 times/week | 25 | 208 928 | 1,140 | 0.99 (0.89-1.11) | 0.97 (0.88-1.09) |  | 26 | | 190 492 | | 1017 | | 0.97 (0.86-1.09) |
| ≥5.0 times/week | 39 | 61 039 | 353 | 1.10 (0.96-1.27) | 1.10 (0.95-1.26) |  | 39 | | 56 665 | | 313 | | 1.06 (0.91-1.23) |
| per 25 g/day |  |  |  | 1.09 (0.96-1.24) | 1.08 (0.95-1.23) |  |  | |  | |  | | 1.06 (0.92-1.22) |
|  |  |  |  | Ptrend=0.181 | Ptrend=0.222 |  |  | |  | |  | | Ptrend=0.442 |
| Fruit |  |  |  |  |  |  |  | |  | |  | |  |
| <2.0 servings/day | 107 | 154 678 | 854 | 1.00 (Ref) | 1.00 (Ref) |  | 107 | | 139 714 | | 755 | | 1.00 (Ref) |
| 2.0-2.9 servings/day | 181 | 118 743 | 631 | 0.94 (0.85-1.04) | 0.96 (0.87-1.07) |  | 181 | | 106 803 | | 561 | | 0.97 (0.87-1.09) |
| 3.0-3.9 servings/day | 231 | 90 574 | 491 | 0.95 (0.85-1.06) | 0.98 (0.87-1.10) |  | 231 | | 80 764 | | 419 | | 0.96 (0.85-1.09) |
| ≥4.0 servings/day | 310 | 103 966 | 581 | 0.95 (0.85-1.05) | 0.99 (0.89-1.10) |  | 311 | | 91 489 | | 497 | | 0.99 (0.88-1.11) |
| per 100 g/day |  |  |  | 0.97 (0.92-1.03) | 1.00 (0.94-1.05) |  |  | |  | |  | | 0.99 (0.94-1.05) |
|  |  |  |  | Ptrend=0.318 | Ptrend=0.855 |  |  | |  | |  | | Ptrend=0.797 |
| Vegetables |  |  |  |  |  |  |  | |  | |  | |  |
| <2.0 servings/day | 162 | 161 306 | 866 | 1.00 (Ref) | 1.00 (Ref) |  | 162 | | 144 801 | | 749 | | 1.00 (Ref) |
| 2.0-2.9 servings/day | 216 | 157 007 | 858 | 0.98 (0.89-1.08) | 0.98 (0.89-1.08) |  | 215 | | 141 194 | | 761 | | 1.01 (0.91-1.11) |
| 3.0-3.9 servings/day | 250 | 82 657 | 466 | 1.01 (0.90-1.13) | 1.01 (0.90-1.13) |  | 250 | | 73 734 | | 407 | | 1.02 (0.90-1.16) |
| ≥4.0 servings/day | 292 | 64 201 | 356 | 1.03 (0.91-1.16) | 1.02 (0.90-1.16) |  | 291 | | 56 699 | | 307 | | 1.03 (0.90-1.18) |
| per 100 g/day |  |  |  | 1.02 (0.93-1.11) | 1.01 (0.93-1.11) |  |  | |  | |  | | 1.03 (0.93-1.13) |
|  |  |  |  | Ptrend=0.699 | Ptrend=0.750 |  |  | |  | |  | | Ptrend=0.605 |
| Fibre |  |  |  |  |  |  |  | |  | |  | |  |
| Lowest fifth | 12.9 | 91 213 | 487 | 1.00 (ref) | 1.00 (ref) |  | 12.8 | | 81 815 | | 425 | | 1.00 (ref) |
| 2 | 14.9 | 90 766 | 501 | 0.98 (0.86-1.11) | 0.99 (0.88-1.13) |  | 14.8 | | 81 783 | | 451 | | 1.03 (0.90-1.17) |
| 3 | 16.2 | 90 396 | 489 | 0.93 (0.82-1.05) | 0.96 (0.84-1.09) |  | 16.1 | | 81 314 | | 421 | | 0.95 (0.83-1.09) |
| 4 | 17.4 | 90 481 | 481 | 0.89 (0.78-1.01) | 0.93 (0.81-1.06) |  | 17.4 | | 81 200 | | 422 | | 0.94 (0.82-1.08) |
| Highest fifth | 19.6 | 90 288 | 499 | 0.89 (0.79-1.01) | 0.94 (0.83-1.07) |  | 19.6 | | 80 111 | | 425 | | 0.94 (0.82-1.08) |
| per 5 g/day |  |  |  | 0.91 (0.83-0.99) | 0.94 (0.86-1.03) |  |  | |  | |  | | 0.94 (0.85-1.03) |
|  |  |  |  | Ptrend=0.030 | Ptrend=0.221 |  |  | |  | |  | | Ptrend=0.198 |
| Alcohol |  |  |  |  |  |  |  | |  | |  | |  |
| <1.0 g/day | 2 | 92 768 | 444 | 1.00 (ref) | 1.00 (ref) |  | 2 | | 76 029 | | 361 | | 1.00 (ref) |
| 1.0-7.9 g/day | 7 | 119 993 | 559 | 1.01 (0.89-1.15) | 1.02 (0.90-1.16) |  | 7 | | 107 836 | | 479 | | 0.98 (0.85-1.12) |
| 8.0-15.9 g/day | 15 | 104 849 | 531 | 1.05 (0.92-1.19) | 1.06 (0.93-1.20) |  | 15 | | 96 234 | | 476 | | 1.04 (0.90-1.19) |
| ≥16.0 g/day | 32 | 156 528 | 1065 | 1.28 (1.14-1.44) | 1.24 (1.10-1.40) |  | 32 | | 143 824 | | 951 | | 1.21 (1.07-1.38) |
| per 10 g/day |  |  |  | 1.09 (1.06-1.13) | 1.08 (1.04-1.12) |  |  | |  | |  | | 1.08 (1.04-1.12) |
|  |  |  |  | Ptrend<0.001 | Ptrend<0.001 |  |  | |  | |  | | Ptrend<0.001 |
| Tea |  |  |  |  |  |  |  | |  | |  | |  |
| <2.0 cups/day | 147 | 124 710 | 658 | 1.00 (ref) | 1.00 (ref) |  | 146 | | 111 413 | | 566 | | 1.00 (ref) |
| 2.0-3.9 cups/day | 460 | 139 179 | 813 | 1.01 (0.91-1.12) | 1.03 (0.93-1.14) |  | 460 | | 125 211 | | 720 | | 1.05 (0.94-1.17) |
| 4.0-5.9 cups/day | 679 | 120 345 | 638 | 0.90 (0.80-1.00) | 0.92 (0.83-1.03) |  | 679 | | 107 848 | | 558 | | 0.94 (0.83-1.05) |
| ≥6.0 cups/day | 880 | 89 260 | 485 | 0.92 (0.82-1.04) | 0.95 (0.85-1.07) |  | 880 | | 78 952 | | 420 | | 0.97 (0.85-1.10) |
| per 200 mL/day |  |  |  | 0.97 (0.94-1.00) | 0.98 (0.95-1.01) |  |  | |  | |  | | 0.98 (0.95-1.02) |
|  |  |  |  | Ptrend=0.050 | Ptrend=0.165 |  |  | |  | |  | | Ptrend=0.295 |
| Coffee |  |  |  |  |  |  |  | |  | |  | |  |
| 0 cups/day | 48 | 105 274 | 564 | 1.00 (ref) | 1.00 (ref) |  | 47 | | 91 176 | | 468 | | 1.00 (ref) |
| 0.5-1.9 cup/day | 188 | 128 851 | 694 | 0.91 (0.82-1.02) | 0.91 (0.81-1.01) |  | 187 | | 115 242 | | 611 | | 0.93 (0.82-1.05) |
| 2.0-2.9 cups/day | 348 | 88 681 | 509 | 0.95 (0.85-1.08) | 0.93 (0.83-1.05) |  | 347 | | 80 457 | | 447 | | 0.94 (0.83-1.08) |
| ≥3.0 cups/day | 584 | 150 598 | 827 | 0.94 (0.84-1.04) | 0.90 (0.81-1.00) |  | 583 | | 136 505 | | 737 | | 0.92 (0.81-1.04) |
| per 200 mL/day |  |  |  | 0.99 (0.95-1.03) | 0.97 (0.94-1.01) |  |  | |  | |  | | 0.98 (0.94-1.02) |
|  |  |  |  | Ptrend=0.521 | Ptrend=0.146 |  |  | |  | |  | | Ptrend=0.291 |
| CI: confidence interval HR: hazard ratio | | | | | | | | | | | | | |
| ^*^Excludes 49,072 participants who reported changing their diet due to illness and 1,399 who preferred not to answer this question (a total of 336 incident colorectal cancer cases excluded) | | | | | | | | | | | | | |
| ^†^Mean intake from participants who completed at least one 24-hour dietary assessment. For participants who completed more than one 24-hour dietary assessment, their mean intake was taken first, before taking the mean of the group | | | | | | | | | | | | | |
| ^‡^Minimally adjusted model is stratified by age category (5 year categories), deprivation (Townsend score, quintiles), sex and region (10 regions) | | | | | | | | | | | | | |
| ^§^Fully adjusted model is stratified by age category (5 year categories), deprivation (Townsend score, quintiles), sex and region (10 regions), and adjusted for waist circumference (sex-specific quintiles), height (sex-specific quintiles), smoking (never, previous, current < 15 cigarettes/day, current 15 or more cigarettes per day, unknown), alcohol (<1 g/d, 1-7 g/d, 8-15 g/d, ≥16 g/d, unknown), education (College or University degree, vocational qualifications (other professional qualifications/NVQ or HND or HNC), optional national exams at ages 17 to 18 years (A levels/AS levels), national exams at age 16 years (O levels/GCSEs/CSEs), none of the above, unknown) , physical activity (low: < 10 excess MET-hours per week, moderate: 10-49.9 excess MET-hrs per week, high 50+ excess MET-hours per week, unknown) , family history of CRC (mother, father, or sibling with colorectal cancer), regular NSAID use (regular asprin or Ibuprofen use), regular Vit D supplement use, regular folate supplement use, and in women only: parity (0, 1-2, ≥3 live births, unknown), menopause status (pre-menopausal, post-menopausal, not sure - had a hysterectomy, not sure other reason, unknown), ever OCA use (never, ever, unknown), and ever HRT use (never, ever, unknown) | | | | | | | | | | | | | |

| **Supplementary Table 4** Associations between dietary factors and colorectal cancer in men and women in UK Biobank | | | | | | | | | | | | | | | | |  |
| --- | --- | --- | --- | --- | --- | --- | --- | --- | --- | --- | --- | --- | --- | --- | --- | --- | --- |
|  | **Men** | | | | | | | |  | **Women** | | | | | | | Heterogeneity by sex^‡^ |
| Reported consumption at recruitment | Mean intake (g/day)* | | Participants | | Cases | | HR (95% CI) from fully adjusted model^†^ | |  | Mean intake (g/day) | Participants | | Cases | | HR (95% CI) from fully adjusted model^†^ | |  |
| Red and processed meat |  | |  | |  | |  | |  |  |  | |  | |  | |  |
| <2.0 times/week | 22 | | 19 769 | | 90 | | 1.00 (Ref) | |  | 21 | 48 590 | | 184 | | 1.00 (Ref) | |  |
| 2.0-2.9 times/week | 57 | | 51 350 | | 316 | | 1.16 (0.92-1.47) | |  | 49 | 84 623 | | 388 | | 1.10 (0.93-1.32) | |  |
| 3.0-3.9 times/week | 69 | | 30 847 | | 213 | | 1.24 (0.97-1.59) | |  | 60 | 40 544 | | 175 | | 1.00 (0.81-1.23) | |  |
| ≥4.0 times/week | 81 | | 113 662 | | 866 | | 1.41 (1.13-1.75) | |  | 68 | 78 938 | | 343 | | 1.01 (0.85-1.21) | |  |
| per 50 g/day |  | |  | |  | | 1.39 (1.17-1.64) | |  |  |  | |  | | 0.99 (0.83-1.19) | |  |
|  |  | |  | |  | | Ptrend<0.001 | |  |  |  | |  | | Ptrend=0.941 | | P=0.008 |
| Red meat |  | |  | |  | |  | |  |  |  | |  | |  | |  |
| <1.0 time/week | 9 | | 15 789 | | 67 | | 1.00 (Ref) | |  | 7 | 32 006 | | 120 | | 1.00 (Ref) | |  |
| 1.0-1.9 times/week | 38 | | 81 577 | | 513 | | 1.26 (0.97-1.62) | |  | 31 | 103 239 | | 434 | | 1.00 (0.82-1.23) | |  |
| 2.0-2.9 times/week | 47 | | 63 138 | | 454 | | 1.34 (1.04-1.74) | |  | 41 | 68 348 | | 327 | | 1.07 (0.86-1.32) | |  |
| ≥3.0 times/week | 58 | | 55 388 | | 452 | | 1.47 (1.13-1.90) | |  | 50 | 49 425 | | 209 | | 0.90 (0.72-1.13) | |  |
| per 50 g/day |  | |  | |  | | 1.47 (1.17-1.84) | |  |  |  | |  | | 0.94 (0.73-1.19) | |  |
|  |  | |  | |  | | Ptrend=0.001 | |  |  |  | |  | | Ptrend=0.590 | | P=0.008 |
| Processed meat |  | |  | |  | |  | |  |  |  | |  | |  | |  |
| Never | 5 | | 11 875 | | 50 | | 1.00 (Ref) | |  | 5 | 32 232 | | 125 | | 1.00 (Ref) | |  |
| <1.0 time/week | 18 | | 46 627 | | 291 | | 1.22 (0.90-1.65) | |  | 15 | 97 046 | | 437 | | 1.06 (0.87-1.30) | |  |
| 1.0 time/week | 24 | | 65 038 | | 462 | | 1.38 (1.03-1.85) | |  | 20 | 73 201 | | 319 | | 1.03 (0.83-1.27) | |  |
| ≥2.0 times/week | 32 | | 94 711 | | 695 | | 1.47 (1.10-1.97) | |  | 25 | 52 706 | | 218 | | 1.03 (0.79-1.24) | |  |
| per 25 g/day |  | |  | |  | | 1.39 (1.15-1.68) | |  |  |  | |  | | 0.97 (0.75-1.25) | |  |
|  |  | |  | |  | | Ptrend=0.001 | |  |  |  | |  | | Ptrend=0.787 | | P=0.022 |
| Poultry |  | |  | |  | |  | |  |  |  | |  | |  | |  |
| Never | 2 | | 8 525 | | 43 | | 1.00 (Ref) | |  | 2 | 15 803 | | 65 | | 1.00 (Ref) | |  |
| <1.0 time/week | 19 | | 24 663 | | 159 | | 0.96 (0.69-1.35) | |  | 19 | 26 138 | | 124 | | 0.88 (0.65-1.19) | |  |
| 1.0 time/week | 28 | | 80 665 | | 613 | | 1.17 (0.86-1.60) | |  | 29 | 89 021 | | 403 | | 0.85 (0.66-1.11) | |  |
| ≥2.0 times/week | 40 | | 104 407 | | 682 | | 1.14 (0.83-1.55) | |  | 40 | 124 377 | | 506 | | 0.83 (0.64-1.08) | |  |
| per 25 g/day |  | |  | |  | | 1.11 (0.96-1.27) | |  |  |  | |  | | 0.90 (0.78-1.05) | |  |
|  |  | |  | |  | | Ptrend=0.166 | |  |  |  | |  | | Ptrend=0.182 | | P=0.054 |
| Total fish |  | |  | |  | |  | |  |  |  | |  | |  | |  |
| <1.0 time/week | 6 | | 16 709 | | 89 | | 1.00 (Ref) | |  | 6 | 19 803 | | 76 | | 1.00 (Ref) | |  |
| 1.0-1.9 times/week | 21 | | 90 794 | | 617 | | 1.07 (0.85-1.34) | |  | 22 | 98 140 | | 390 | | 0.88 (0.69-1.13) | |  |
| 2.0-2.9 times/week | 29 | | 46 558 | | 358 | | 1.03 (0.81-1.30) | |  | 29 | 61 817 | | 285 | | 0.92 (0.72-1.19) | |  |
| ≥3.0 times/week | 40 | | 59 275 | | 422 | | 1.01 (0.81-1.28) | |  | 37 | 73 995 | | 339 | | 0.89 (0.69-1.15) | |  |
| per 25 g/day |  | |  | |  | | 0.96 (0.84-1.10) | |  |  |  | |  | | 0.95 (0.80-1.14) | |  |
|  |  | |  | |  | | Ptrend=0.547 | |  |  |  | |  | | Ptrend=0.608 | | P=0.960 |
| Dairy milk |  | |  | |  | |  | |  |  |  | |  | |  | |  |
| never | 30 | | 6 857 | | 39 | | 1.00 (Ref) | |  | 30 | 8 966 | | 46 | | 1.00 (Ref) | |  |
| <150 mL/day | 158 | | 38 414 | | 285 | | 1.40 (1.00-1.96) | |  | 156 | 46 293 | | 181 | | 0.82 (0.59-1.14) | |  |
| 150-299 mL/day | 225 | | 115 874 | | 805 | | 1.18 (0.85-1.63) | |  | 221 | 133 519 | | 599 | | 0.83 (0.61-1.12) | |  |
| ≥300 mL/day | 283 | | 49 505 | | 327 | | 1.13 (0.81-1.58) | |  | 276 | 50 304 | | 213 | | 0.77 (0.56-1.06) | |  |
| per 100 mL/day |  | |  | |  | | 0.94 (0.86-1.04) | |  |  |  | |  | | 0.92 (0.81-1.03) | | P=0.770 |
|  |  | |  | |  | | Ptrend=0.225 | |  |  |  | |  | | Ptrend=0.161 | |  |
| Cheese |  | |  | |  | |  | |  |  |  | |  | |  | |  |
| <1.0 time/week | 12 | | 37 291 | | 258 | | 1.00 (ref) | |  | 14 | 55 603 | | 240 | | 1.00 (ref) | |  |
| 1.0 time/week | 18 | | 44 238 | | 313 | | 1.00 (0.85-1.19) | |  | 19 | 55 027 | | 228 | | 0.95 (0.79-1.14) | |  |
| 2.0-4.9 times/week | 26 | | 101 715 | | 690 | | 0.96 (0.83-1.11) | |  | 25 | 107 213 | | 450 | | 0.99 (0.84-1.16) | |  |
| ≥5.0 times/week | 41 | | 29 975 | | 203 | | 1.01 (0.84-1.22) | |  | 37 | 31 064 | | 150 | | 1.22 (0.99-1.50) | |  |
| per 25 g/day |  | |  | |  | | 1.00 (0.86-1.16) | |  |  |  | |  | | 1.23 (0.99-1.53) | |  |
|  |  | |  | |  | | Ptrend=0.960 | |  |  |  | |  | | Ptrend=0.064 | | P=0.124 |
| Fruit |  | |  | |  | |  | |  |  |  | |  | |  | |  |
| <2.0 servings/day | 103 | | 86 409 | | 582 | | 1.00 (Ref) | |  | 112 | 68 269 | | 272 | | 1.00 (Ref) | |  |
| 2.0-2.9 servings/day | 182 | | 53 540 | | 359 | | 0.96 (0.84-1.09) | |  | 180 | 65 203 | | 272 | | 0.97 (0.82-1.15) | |  |
| 3.0-3.9 servings/day | 232 | | 35 800 | | 246 | | 0.97 (0.83-1.13) | |  | 230 | 54 774 | | 245 | | 0.99 (0.83-1.18) | |  |
| ≥4.0 servings/day | 314 | | 39 751 | | 282 | | 1.00 (0.86-1.15) | |  | 308 | 64 215 | | 299 | | 0.99 (0.84-1.17) | |  |
| per 100 g/day |  | |  | |  | | 0.99 (0.93-1.06) | |  |  |  | |  | | 1.00 (0.92-1.08) | |  |
|  |  | |  | |  | | Ptrend=0.862 | |  |  |  | |  | | Ptrend=0.972 | | P=0.910 |
| Vegetables |  | |  | |  | |  | |  |  |  | |  | |  | |  |
| <2.0 servings/day | 150 | | 84 895 | | 549 | | 1.00 (Ref) | |  | 175 | 76 411 | | 317 | | 1.00 (Ref) | |  |
| 2.0-2.9 servings/day | 196 | | 68 342 | | 475 | | 0.99 (0.87-1.12) | |  | 229 | 88 655 | | 383 | | 0.98 (0.84-1.14) | |  |
| 3.0-3.9 servings/day | 228 | | 33 261 | | 266 | | 1.10 (0.95-1.27) | |  | 264 | 49 396 | | 200 | | 0.91 (0.76-1.09) | |  |
| ≥4.0 servings/day | 267 | | 26 847 | | 173 | | 0.92 (0.77-1.09) | |  | 307 | 37 354 | | 183 | | 1.15 (0.95-1.38) | |  |
| per 100 g/day |  | |  | |  | | 0.99 (0.87-1.12) | |  |  |  | |  | | 1.06 (0.92-1.21) | |  |
|  |  | |  | |  | | Ptrend=0.842 | |  |  |  | |  | | Ptrend=0.430 | | P=0.444 |
| Fibre |  | |  | |  | |  | |  |  |  | |  | |  | |  |
| Lowest fifth | 13.1 | | 49 128 | | 322 | | 1.00 (ref) | |  | 12.6 | 42 085 | | 165 | | 1.00 (ref) | |  |
| 2 | 15.1 | | 41 634 | | 308 | | 1.05 (0.90-1.23) | |  | 14.6 | 49 132 | | 193 | | 0.91 (0.74-1.13) | |  |
| 3 | 16.5 | | 38 388 | | 265 | | 0.96 (0.81-1.13) | |  | 15.9 | 52 008 | | 224 | | 0.96 (0.78-1.18) | |  |
| 4 | 17.8 | | 37 833 | | 245 | | 0.89 (0.75-1.05) | |  | 17.1 | 52 648 | | 236 | | 0.97 (0.79-1.19) | |  |
| Highest fifth | 20.2 | | 40 928 | | 268 | | 0.91 (0.77-1.07) | |  | 19.1 | 49 360 | | 231 | | 0.98 (0.80-1.21) | |  |
| per 5 g/day |  | |  | |  | | 0.91 (0.81-1.01) | |  |  |  | |  | | 1.01 (0.88-1.17) | |  |
|  |  | |  | |  | | Ptrend=0.082 | |  |  |  | |  | | Ptrend=0.868 | | P=0.217 |
| Alcohol |  | |  | |  | |  | |  |  |  | |  | |  | |  |
| <1.0 g/day | 2 | | 28 819 | | 153 | | 1.00 (ref) | |  | 1 | 63 949 | | 291 | | 1.00 (ref) | |  |
| 1.0-7.9 g/day | 7 | | 38 510 | | 205 | | 1.03 (0.84-1.28) | |  | 7 | 81 483 | | 354 | | 1.02 (0.87-1.19) | |  |
| 8.0-15.9 g/day | 15 | | 42 996 | | 281 | | 1.23 (1.01-1.50) | |  | 15 | 61 853 | | 250 | | 0.95 (0.80-1.13) | |  |
| ≥16.0 g/day | 34 | | 108 266 | | 861 | | 1.45 (1.22-1.73) | |  | 28 | 48 262 | | 204 | | 1.01 (0.84-1.21) | |  |
| per 10 g/day |  | |  | |  | | 1.12 (1.08-1.17) | |  |  |  | |  | | 0.99 (0.93-1.06) | |  |
|  |  | |  | |  | | Ptrend<0.001 | |  |  |  | |  | | Ptrend=0.858 | | P=0.002 |
| Tea |  | |  | |  | |  | |  |  |  | |  | |  | |  |
| <2.0 cups/day | 124 | | 57 362 | | 390 | | 1.00 (ref) | |  | 166 | 67 348 | | 268 | | 1.00 (ref) | |  |
| 2.0-3.9 cups/day | 436 | | 64 335 | | 457 | | 0.96 (0.84-1.10) | |  | 480 | 74 844 | | 356 | | 1.15 (0.98-1.34) | |  |
| 4.0-5.9 cups/day | 652 | | 54 502 | | 361 | | 0.88 (0.76-1.01) | |  | 699 | 65 843 | | 277 | | 1.00 (0.84-1.18) | |  |
| ≥6.0 cups/day | 858 | | 42 109 | | 287 | | 0.93 (0.79-1.08) | |  | 899 | 47 151 | | 198 | | 1.01 (0.84-1.21) | |  |
| per 200 mL/day |  | |  | |  | | 0.97 (0.93-1.01) | |  |  |  | |  | | 1.00 (0.95-1.04) | |  |
|  |  | |  | |  | | Ptrend=0.143 | |  |  |  | |  | | Ptrend=0.833 | | P=0.396 |
| Coffee |  | |  | |  | |  | |  |  |  | |  | |  | |  |
| 0 cups/day | 53 | | 44 430 | | 321 | | 1.00 (ref) | |  | 45 | 60 844 | | 243 | | 1.00 (ref) | |  |
| 0.5-1.9 cup/day | 187 | | 56 462 | | 380 | | 0.85 (0.73-0.99) | |  | 188 | 72 389 | | 314 | | 0.97 (0.82-1.15 | |  |
| 2.0-2.9 cups/day | 344 | | 41 122 | | 281 | | 0.85 (0.72-1.00) | |  | 352 | 47 559 | | 228 | | 1.04 (0.86-1.24) | |  |
| ≥3.0 cups/day | 575 | | 76 183 | | 512 | | 0.87 (0.76-1.01) | |  | 592 | 74 415 | | 315 | | 0.91 (0.77-1.08) | |  |
| per 200 mL/day |  | |  | |  | | 0.97 (0.92-1.02) | |  |  |  | |  | | 0.97 (0.92-1.03) | |  |
|  |  | |  | |  | | Ptrend=0.212 | |  |  |  | |  | | Ptrend=0.329 | | P=0.940 |
| CI: confidence interval HR: hazard ratio | | | | | | | | | | | | | | | | | |
| ^*^Mean intake from participants who completed at least one 24-hour dietary assessment. For participants who completed more than one 24-hour dietary assessment, their mean intake was taken first, before taking the mean of the group | | | | | | | | | | | | | | | | | |
| ^†^Fully adjusted model is stratified by age category (5 year categories), deprivation (Townsend score, quintiles), sex and region (10 regions), and adjusted for waist circumference (sex-specific quintiles), height (sex-specific quintiles), smoking (never, previous, current < 15 cigarettes/day, current 15 or more cigarettes per day, unknown), alcohol (<1 g/d, 1-7 g/d, 8-15 g/d, ≥16 g/d, unknown), education (College or University degree, vocational qualifications (other professional qualifications/NVQ or HND or HNC), optional national exams at ages 17 to 18 years (A levels/AS levels), national exams at age 16 years (O levels/GCSEs/CSEs), none of the above, unknown) , physical activity (low: < 10 excess MET-hours per week, moderate: 10-49.9 excess MET-hrs per week, high 50+ excess MET-hours per week, unknown) , family history of CRC (mother, father, or sibling with colorectal cancer), regular NSAID use (regular asprin or Ibuprofen use), regular Vit D supplement use, regular folate supplement use, and in women only: parity (0, 1-2, ≥3 live births, unknown), menopause status (pre-menopausal, post-menopausal, not sure - had a hysterectomy, not sure other reason, unknown), ever OCA use (never, ever, unknown), and ever HRT use (never, ever, unknown) | | | | | | | | | | | | | | | | | |
| ^‡^To test for heterogeneity by sex, we compared models with and without an interaction term for the main exposure (as a continuous trend variable) and sex and evaluated the significance using the likelihood ratio test | | | | | | | | | | | | | | | | | |
|  | |  | |  | |  | |  |  |  | |  | |  | |  |  |

| **Supplementary Table 5** Associations between fish types and colorectal cancer in UK Biobank | | | | | | | | |  | |  | |  | |
| --- | --- | --- | --- | --- | --- | --- | --- | --- | --- | --- | --- | --- | --- | --- |
|  |  | | **All participants (n=475,581)** | | | |  | **Participants who did not report changing their diet due to illness (n=425,112)^*^** | | | | | | |
| Reported consumption at recruitment | | Mean intake (g/day)^†^ | Participants | Cases | HR (95% CI) from minimally adjusted model^‡^ | HR (95% CI) from fully adjusted model^§^ |  | Mean intake (g/day)^†^ | | Participants | | Cases | | HR (95% CI) from fully adjusted model^§^ |
| Oily fish | |  |  |  |  |  |  |  | |  | |  | |  |
| never | | 1 | 52 331 | 263 | 1.00 (Ref) | 1.00 (Ref) |  | 1 | | 46 083 | | 227 | | 1.00 (Ref) |
| <1.0 time/week | | 7 | 156 749 | 830 | 0.95 (0.83-1.09) | 0.93 (0.81-1.07) |  | 7 | | 142 591 | | 726 | | 0.92 (0.79-1.07) |
| 1.0 time/week | | 13 | 177 854 | 994 | 0.91 (0.79-1.04) | 0.89 (0.78-1.02) |  | 13 | | 159 764 | | 874 | | 0.90 (0.77-1.04) |
| ≥2.0 times/week | | 22 | 84 741 | 496 | 0.89 (0.76-1.03) | 0.88 (0.75-1.02) |  | 23 | | 73 409 | | 426 | | 0.89 (0.76-1.05) |
| Per 25 g/day | |  |  |  | 0.87 (0.74-1.02) | 0.87 (0.74-1.02) |  |  | |  | |  | | 0.91 (0.78-1.08) |
|  | |  |  |  | Ptrend=0.082 | Ptrend=0.090 |  |  | |  | |  | | Ptrend=0.278 |
| Non-oily fish | |  |  |  |  |  |  |  | |  | |  | |  |
| never | | 2 | 22 421 | 113 | 1.00 (Ref) | 1.00 (Ref) |  | 2 | | 19 663 | | 96 | | 1.00 (Ref) |
| <1.0 time/week | | 12 | 137 603 | 687 | 0.87 (0.71-1.06) | 0.84 (0.68-1.02) |  | 12 | | 124 275 | | 596 | | 0.83 (0.67-1.03) |
| 1.0 time/week | | 16 | 234 561 | 1,364 | 0.93 (0.76-1.12) | 0.89 (0.73-1.08) |  | 16 | | 210 240 | | 1199 | | 0.90 (0.73-1.12) |
| ≥2.0 times/week | | 23 | 77 442 | 425 | 0.90 (0.73-1.11) | 0.86 (0.70-1.06) |  | 23 | | 68 007 | | 368 | | 0.88 (0.70-1.11) |
| Per 25 g/day | |  |  |  | 0.97 (0.78-1.21) | 0.94 (0.76-1.17) |  |  | |  | |  | | 1.00 (0.79-1.26) |
|  | |  |  |  | Ptrend=0.802 | Ptrend=0.607 |  |  | |  | |  | | Ptrend=0.986 |
| CI: confidence interval HR: hazard ratio | | | | | | | | | | | | | | |
| ^*^Excludes 49,072 participants who reported changing their diet due to illness and 1,399 who preferred not to answer this question (a total of 336 incident colorectal cancer cases excluded) | | | | | | | | | | | | | | |
| ^†^Mean intake from participants who completed at least one 24-hour dietary assessment. For participants who completed more than one 24-hour dietary assessment, their mean intake was taken first, before taking the mean of the group | | | | | | | | | | | | | | |
| ^‡^Minimally adjusted model is stratified by age category (5 year categories), deprivation (Townsend score, quintiles), sex and region (10 regions) | | | | | | | | | | | | | | |
| ^§^Fully adjusted model is stratified by age category (5 year categories), deprivation (Townsend score, quintiles), sex and region (10 regions), and adjusted for waist circumference (sex-specific quintiles), height (sex-specific quintiles), smoking (never, previous, current < 15 cigarettes/day, current 15 or more cigarettes per day, unknown), alcohol (<1 g/d, 1-7 g/d, 8-15 g/d, ≥16 g/d, unknown), education (College or University degree, vocational qualifications (other professional qualifications/NVQ or HND or HNC), optional national exams at ages 17 to 18 years (A levels/AS levels), national exams at age 16 years (O levels/GCSEs/CSEs), none of the above, unknown) , physical activity (low: < 10 excess MET-hours per week, moderate: 10-49.9 excess MET-hrs per week, high 50+ excess MET-hours per week, unknown) , family history of CRC (mother, father, or sibling with colorectal cancer), regular NSAID use (regular asprin or Ibuprofen use), regular Vit D supplement use, regular folate supplement use, and in women only: parity (0, 1-2, ≥3 live births, unknown), menopause status (pre-menopausal, post-menopausal, not sure - had a hysterectomy, not sure other reason, unknown), ever OCA use (never, ever, unknown), and ever HRT use (never, ever, unknown) | | | | | | | | | | | | | | |

| **Supplementary Table 6** Associations between sources of fibre and colorectal cancer in UK Biobank | | | | | | | | |
| --- | --- | --- | --- | --- | --- | --- | --- | --- |
|  | **All participants (n=475,581)** | | | |  | **Participants who did not report changing their diet due to illness (n=425,112)^*^** | | |
| Reported consumption at recruitment | Participants | Cases | HR (95% CI) from minimally adjusted model^†^ | HR (95% CI) from fully adjusted model^‡^ |  | Participants | Cases | HR (95% CI) from fully adjusted model^‡^ |
| Fibre from fruit |  |  |  |  |  |  |  |  |
| Lowest fifth | 121 428 | 668 | 1.00 (ref) | 1.00 (ref) |  | 109 440 | 591 | 1.00 (ref) |
| 2 | 111 550 | 617 | 0.98 (0.88-1.09) | 1.00 (0.90-1.12) |  | 100 237 | 542 | 1.00 (0.89-1.13) |
| 3 | 54 429 | 266 | 0.87 (0.75-1.01) | 0.91 (0.79-1.06) |  | 49 313 | 241 | 0.93 (0.80-1.09) |
| 4 | 92 428 | 509 | 0.96 (0.86-1.08) | 1.00 (0.89-1.13) |  | 82 364 | 432 | 0.98 (0.86-1.12) |
| Highest fifth | 88 126 | 497 | 0.97 (0.86-1.09) | 1.02 (0.90-1.15) |  | 77 416 | 426 | 1.02 (0.90-1.16) |
| Per 5 g/day |  |  | 0.98 (0.92-1.05) | 1.01 (0.95-1.08) |  |  |  | 1.01 (0.94-1.09) |
|  |  |  | Ptrend=0.627 | Ptrend=0.728 |  |  |  | Ptrend=0.788 |
| Fibre from vegetables |  |  |  |  |  |  |  |  |
| Lowest fifth | 156 741 | 841 | 1.00 (ref) | 1.00 (ref) |  | 140 710 | 727 | 1.00 (ref) |
| 2 | 93 869 | 498 | 0.96 (0.86-1.07) | 0.96 (0.85-1.07) |  | 84 683 | 445 | 0.98 (0.87-1.11) |
| 3 | 66 935 | 379 | 1.00 (0.89-1.13) | 1.00 (0.88-1.13) |  | 59 911 | 332 | 1.02 (0.89-1.16) |
| 4 | 83 340 | 472 | 1.01 (0.91-1.14) | 1.01 (0.90-1.13) |  | 74 354 | 413 | 1.03 (0.91-1.16) |
| Highest fifth | 64 286 | 356 | 1.02 (0.90-1.16) | 1.02 (0.90-1.16) |  | 56 770 | 307 | 1.03 (0.90-1.18) |
| Per 5 g/day |  |  | 1.02 (0.95-1.10) | 1.02 (0.95-1.09) |  |  |  | 1.02 (0.95-1.11) |
|  |  |  | Ptrend=0.596 | Ptrend=0.633 |  |  |  | Ptrend=0.575 |
| Fibre from bread and breakfast cereals |  |  |  |  |  |  |  |  |
| Lowest fifth | 93 051 | 487 | 1.00 (ref) | 1.00 (ref) |  | 82 372 | 428 | 1.00 (ref) |
| 2 | 93 233 | 523 | 0.98 (0.87-1.11) | 0.99 (0.87-1.12) |  | 83 171 | 467 | 1.01 (0.88-1.15) |
| 3 | 92 368 | 520 | 0.95 (0.84-1.08) | 0.97 (0.85-1.10) |  | 82 595 | 451 | 0.96 (0.84-1.09) |
| 4 | 92 859 | 498 | 0.87 (0.76-0.98) | 0.89 (0.79-1.01) |  | 83 828 | 427 | 0.86 (0.75-0.99) |
| Highest fifth | 92 692 | 493 | 0.82 (0.72-0.93) | 0.86 (0.76-0.98) |  | 83 637 | 427 | 0.84 (0.73-0.96) |
| Per 5 g/day |  |  | 0.87 (0.81-0.94) | 0.90 (0.83-0.97) |  |  |  | 0.88 (0.81-0.95) |
|  |  |  | Ptrend<0.001 | Ptrend=0.005 |  |  |  | Ptrend=0.002 |
| CI: confidence interval HR: hazard ratio | | | | | | | | |
| ^*^Excludes 49,072 participants who reported changing their diet due to illness and 1,399 who preferred not to answer this question (a total of 336 incident colorectal cancer cases excluded) | | | | | | | | |
| ^†^Mean intake from participants who completed at least one 24-hour dietary assessment. For participants who completed more than one 24-hour dietary assessment, their mean intake was taken first, before taking the mean of the group | | | | | | | | |
| ^‡^Minimally adjusted model is stratified by age category (5 year categories), deprivation (Townsend score, quintiles), sex and region (10 regions) | | | | | | | | |
| ^§^Fully adjusted model is stratified by age category (5 year categories), deprivation (Townsend score, quintiles), sex and region (10 regions), and adjusted for waist circumference (sex-specific quintiles), height (sex-specific quintiles), smoking (never, previous, current < 15 cigarettes/day, current 15 or more cigarettes per day, unknown), alcohol (<1 g/d, 1-7 g/d, 8-15 g/d, ≥16 g/d, unknown), education (College or University degree, vocational qualifications (other professional qualifications/NVQ or HND or HNC), optional national exams at ages 17 to 18 years (A levels/AS levels), national exams at age 16 years (O levels/GCSEs/CSEs), none of the above, unknown) , physical activity (low: < 10 excess MET-hours per week, moderate: 10-49.9 excess MET-hrs per week, high 50+ excess MET-hours per week, unknown) , family history of CRC (mother, father, or sibling with colorectal cancer), regular NSAID use (regular asprin or Ibuprofen use), regular Vit D supplement use, regular folate supplement use, and in women only: parity (0, 1-2, ≥3 live births, unknown), menopause status (pre-menopausal, post-menopausal, not sure - had a hysterectomy, not sure other reason, unknown), ever OCA use (never, ever, unknown), and ever HRT use (never, ever, unknown) | | | | | | | | |

| **Supplementary Table 7** Associations between alcohol types and colorectal cancer in UK Biobank | | | | | | | |  | |  | |  | |
| --- | --- | --- | --- | --- | --- | --- | --- | --- | --- | --- | --- | --- | --- |
|  |  | **All participants (n=475,581)** | | | |  | **Participants who did not report changing their diet due to illness (n=425,112)^*^** | | | | | | |
| Reported consumption at recruitment | Mean intake (g/day)^†^ | Participants | Cases | HR (95% CI) from minimally adjusted model^‡^ | HR (95% CI) from fully adjusted model^§^ |  | Mean intake (g/day)^†^ | | Participants | | Cases | | HR (95% CI) from fully adjusted model^§^ |
| Alcohol from beer |  |  |  |  |  |  |  | |  | |  | |  |
| <1.0g/day | 0.9 | 182 310 | 884 | 1.00 (ref) | 1.00 (ref) |  | 0.9 | | 166 520 | | 785 | | 1.00 (ref) |
| 1.0-7.9 g/day | 4.3 | 80 370 | 435 | 1.03 (0.91-1.16) | 1.02 (0.90-1.16) |  | 4.3 | | 73 539 | | 395 | | 1.07 (0.94-1.22) |
| 8.0-15.9 g/day | 10.5 | 44 053 | 247 | 0.98 (0.84-1.15) | 0.96 (0.82-1.12) |  | 10.5 | | 40 099 | | 215 | | 0.97 (0.82-1.15) |
| ≥16.0 g/day | 25.8 | 58 677 | 475 | 1.36 (1.18-1.56) | 1.30 (1.13-1.50) |  | 25.8 | | 53 151 | | 419 | | 1.33 (1.15-1.55) |
| per 10 g/day |  |  |  | 1.14 (1.08-1.20) | 1.11 (1.06-1.18) |  |  | |  | |  | | 1.12 (1.06-1.19) |
|  |  |  |  | Ptrend<0.001 | Ptrend<0.001 |  |  | |  | |  | | Ptrend<0.001 |
| Alcohol from wine |  |  |  |  |  |  |  | |  | |  | |  |
| <1.0g/day | 1.6 | 85 073 | 480 | 1.00 (ref) | 1.00 (ref) |  | 1.6 | | 74 428 | | 419 | | 1.00 (ref) |
| 1.0-7.9 g/day | 6.8 | 127 025 | 663 | 0.92 (0.81-1.04) | 0.95 (0.84-1.07) |  | 6.8 | | 116 031 | | 575 | | 0.90 (0.78-1.02) |
| 8.0-15.0 g/day | 14.8 | 89 070 | 477 | 0.93 (0.82-1.06) | 0.95 (0.83-1.09) |  | 14.8 | | 82 854 | | 432 | | 0.92 (0.80-1.06) |
| ≥16.0 g/day | 28.4 | 62 205 | 407 | 1.13 (0.98-1.29) | 1.11 (0.96-1.28) |  | 28.5 | | 58 244 | | 376 | | 1.09 (0.94-1.27) |
| per 10 g/day |  |  |  | 1.06 (1.01-1.11) | 1.05 (1.00-1.10) |  |  | |  | |  | | 1.05 (1.00-1.11) |
|  |  |  |  | Ptrend=0.022 | Ptrend=0.063 |  |  | |  | |  | | Ptrend=0.049 |
| Alcohol from spirits |  |  |  |  |  |  |  | |  | |  | |  |
| <1.0g/day | 0.3 | 238 761 | 1,243 | 1.00 (ref) | 1.00 (ref) |  | 0.3 | | 218 114 | | 1106 | | 1.00 (ref) |
| 1.0-7.9 g/day | 1.5 | 95 881 | 544 | 0.95 (0.86-1.05) | 0.94 (0.85-1.04) |  | 1.5 | | 87 631 | | 487 | | 0.95 (0.85-1.05) |
| 8.0-15.9 g/day | 4.7 | 17 968 | 142 | 1.19 (1.00-1.42) | 1.12 (0.94-1.34) |  | 4.7 | | 16 225 | | 126 | | 1.14 (0.95-1.38) |
| ≥16.0 g/day | 11.0 | 11 377 | 92 | 1.13 (0.91-1.40) | 1.05 (0.84-1.30) |  | 10.9 | | 10 128 | | 79 | | 1.04 (0.82-1.31) |
| per 10 g/day |  |  |  | 1.17 (0.97-1.41) | 1.08 (0.90-1.31) |  |  | |  | |  | | 1.08 (0.88-1.32) |
|  |  |  |  | Ptrend=0.096 | Ptrend=0.417 |  |  | |  | |  | | Ptrend=0.454 |
| CI: confidence interval HR: hazard ratio | | | | | | | | | | | | | |
| ^*^Excludes 49,072 participants who reported changing their diet due to illness and 1,399 who preferred not to answer this question (a total of 336 incident colorectal cancer cases excluded) | | | | | | | | | | | | | |
| ^†^Mean intake from participants who completed at least one 24-hour dietary assessment. For participants who completed more than one 24-hour dietary assessment, their mean intake was taken first, before taking the mean of the group | | | | | | | | | | | | | |
| ^‡^Minimally adjusted model is stratified by age category (5 year categories), deprivation (Townsend score, quintiles), sex and region (10 regions) | | | | | | | | | | | | | |
| ^§^Fully adjusted model is stratified by age category (5 year categories), deprivation (Townsend score, quintiles), sex and region (10 regions), and adjusted for waist circumference (sex-specific quintiles), height (sex-specific quintiles), smoking (never, previous, current < 15 cigarettes/day, current 15 or more cigarettes per day, unknown), education (College or University degree, vocational qualifications (other professional qualifications/NVQ or HND or HNC), optional national exams at ages 17 to 18 years (A levels/AS levels), national exams at age 16 years (O levels/GCSEs/CSEs), none of the above, unknown) , physical activity (low: < 10 excess MET-hours per week, moderate: 10-49.9 excess MET-hrs per week, high 50+ excess MET-hours per week, unknown) , family history of CRC (mother, father, or sibling with colorectal cancer), regular NSAID use (regular asprin or Ibuprofen use), regular Vit D supplement use, regular folate supplement use, and in women only: parity (0, 1-2, ≥3 live births, unknown), menopause status (pre-menopausal, post-menopausal, not sure - had a hysterectomy, not sure other reason, unknown), ever OCA use (never, ever, unknown), and ever HRT use (never, ever, unknown) | | | | | | | | | | | | | |

| **Supplementary Table 8** Associations between dietary factors and colon and rectal cancer in UK Biobank | | | | | | | |  |
| --- | --- | --- | --- | --- | --- | --- | --- | --- |
| Reported consumption at recruitment |  |  | **Colon** | |  | **Rectal** | |  |
|  | Mean intake (g/day)* | Participants | Cases | HR (95% CI) from fully adjusted model^†^ |  | Cases | HR (95% CI) from fully adjusted model^†^ | Heterogeneity by subsite^‡^ |
| Red and processed meat |  |  |  |  |  |  |  |  |
| <2.0 times/week | 21 | 68 359 | 186 | 1.00 (Ref) |  | 88 | 1.00 (Ref) |  |
| 2.0-2.9 times/week | 52 | 135 973 | 477 | 1.12 (0.94-1.33) |  | 227 | 1.08 (0.84-1.38) |  |
| 3.0-3.9 times/week | 64 | 71 391 | 274 | 1.15 (0.95-1.39) |  | 114 | 0.95 (0.72-1.27) |  |
| ≥4.0 times/week | 76 | 192 600 | 781 | 1.19 (1.01-1.41) |  | 428 | 1.20 (0.94-1.52) |  |
| per 50 g/day |  |  |  | 1.17 (1.01-1.35) |  |  | 1.18 (0.96-1.45) |  |
|  |  |  |  | Ptrend=0.032 |  |  | Ptrend=0.113 | P=0.932 |
| Red meat |  |  |  |  |  |  |  |  |
| <1 time/week | 8 | 47 795 | 123 | 1.00 (Ref) |  | 64 | 1.00 (Ref) |  |
| 1.0-1.9 times/week | 34 | 184 816 | 623 | 1.11 (0.91-1.34) |  | 324 | 1.05 (0.80-1.39) |  |
| 2.0-2.9 times/week | 44 | 131 486 | 528 | 1.22 (1.00-1.49) |  | 253 | 1.05 (0.79-1.39) |  |
| ≥3.0 times/week | 54 | 104 813 | 444 | 1.21 (0.99-1.48) |  | 217 | 1.05 (0.79-1.40) |  |
| per 50 g/day |  |  |  | 1.26 (1.03-1.55) |  |  | 1.04 (0.78-1.38) |  |
|  |  |  |  | Ptrend=0.025 |  |  | Ptrend=0.808 | P=0.272 |
| Processed meat |  |  |  |  |  |  |  |  |
| Never | 5 | 44 107 | 122 | 1.00 (Ref) |  | 53 | 1.00 (Ref) |  |
| <1.0 time/week | 16 | 143 673 | 506 | 1.10 (0.90-1.34) |  | 222 | 1.08 (0.80-1.46) |  |
| 1.0 time/week | 22 | 138 239 | 538 | 1.16 (0.95-1.42) |  | 243 | 1.09 (0.80-1.48) |  |
| ≥2.0 times/week | 29 | 147 417 | 568 | 1.12 (0.92-1.38) |  | 345 | 1.32 (0.98-1.78) |  |
| per 25 g/day |  |  |  | 1.10 (0.92-1.32) |  |  | 1.41 (1.08-1.83) |  |
|  |  |  |  | Ptrend=0.294 |  |  | Ptrend=0.011 | P=0.132 |
| Poultry |  |  |  |  |  |  |  |  |
| Never | 2 | 24 328 | 75 | 1.00 (Ref) |  | 33 | 1.00 (Ref) |  |
| <1.0 time/week | 19 | 50 801 | 188 | 0.86 (0.65-1.12) |  | 95 | 0.96 (0.64-1.43) |  |
| 1.0 time/week | 28 | 169 686 | 668 | 0.94 (0.74-1.20) |  | 348 | 1.09 (0.76-1.56) |  |
| ≥2.0 times/week | 40 | 228 784 | 802 | 0.95 (0.74-1.20) |  | 386 | 1.01 (0.70-1.45) |  |
| per 25 g/day |  |  |  | 1.02 (0.90-1.15) |  |  | 0.99 (0.83-1.19) |  |
|  |  |  |  | Ptrend=0.779 |  |  | Ptrend=0.946 | P=0.828 |
| Total fish |  |  |  |  |  |  |  |  |
| <1.0 time/week | 6 | 36 512 | 113 | 1.00 (Ref) |  | 52 | 1.00 (Ref) |  |
| 1.0-1.9 times/week | 21 | 188 934 | 685 | 0.98 (0.80-1.20) |  | 322 | 0.99 (0.74-1.34) |  |
| 2.0-2.9 times/week | 29 | 111 375 | 419 | 0.92 (0.75-1.14) |  | 224 | 1.09 (0.80-1.48) |  |
| ≥3.0 times/week | 39 | 133 270 | 504 | 0.92 (0.75-1.13) |  | 257 | 1.03 (0.76-1.40) |  |
| per 25 g/day |  |  |  | 0.92 (0.81-1.05) |  |  | 1.04 (0.87-1.26) |  |
|  |  |  |  | Ptrend=0.221 |  |  | Ptrend=0.632 | P=0.274 |
| Dairy milk |  |  |  |  |  |  |  |  |
| never | 30 | 15 823 | 59 | 1.00 (Ref) |  | 26 | 1.00 (Ref) |  |
| <150 mL/day | 157 | 84 707 | 298 | 1.00 (0.76-1.33) |  | 168 | 1.31 (0.86-1.98) |  |
| 150-299 mL/day | 223 | 249 393 | 972 | 0.98 (0.75-1.27) |  | 432 | 1.00 (0.67-1.49) |  |
| ≥300 mL/day | 279 | 99 809 | 335 | 0.84 (0.64-1.11) |  | 205 | 1.15 (0.76-1.73) |  |
| per 100 mL/day |  |  |  | 0.92 (0.84-1.01) |  |  | 0.97 (0.85-1.10) |  |
|  |  |  |  | Ptrend=0.065 |  |  | Ptrend=0.605 | P=0.529 |
| Cheese |  |  |  |  |  |  |  |  |
| <1.0 time/week | 13 | 92 894 | 352 | 1.00 (ref) |  | 146 | 1.00 (ref) |  |
| 1.0 time/week | 18 | 99 265 | 362 | 0.94 (0.81-1.09) |  | 179 | 1.09 (0.87-1.35) |  |
| 2.0-4.9 times/week | 25 | 208 928 | 747 | 0.92 (0.81-1.05) |  | 393 | 1.10 (0.91-1.33) |  |
| ≥5.0 times/week | 39 | 61 039 | 235 | 1.05 (0.89-1.25) |  | 118 | 1.20 (0.94-1.54) |  |
| per 25 g/day |  |  |  | 1.04 (0.89-1.22) |  |  | 1.17 (0.94-1.46) |  |
|  |  |  |  | Ptrend=0.601 |  |  | Ptrend=0.167 | P=0.410 |
| Fruit |  |  |  |  |  |  |  |  |
| <2.0 servings/day | 107 | 154 678 | 559 | 1.00 (Ref) |  | 295 | 1.00 (Ref) |  |
| 2.0-2.9 servings/day | 181 | 118 743 | 410 | 0.94 (0.82-1.07) |  | 221 | 1.01 (0.84-1.20) |  |
| 3.0-3.9 servings/day | 231 | 90 574 | 331 | 0.98 (0.85-1.12) |  | 160 | 0.98 (0.80-1.19) |  |
| ≥4.0 servings/day | 310 | 103 966 | 406 | 1.02 (0.89-1.16) |  | 175 | 0.93 (0.76-1.13) |  |
| per 100 g/day |  |  |  | 1.01 (0.95-1.08) |  |  | 0.96 (0.88-1.06) |  |
|  |  |  |  | Ptrend=0.753 |  |  | Ptrend=0.447 | P=0.422 |
| Vegetables |  |  |  |  |  |  |  |  |
| <2.0 servings/day | 162 | 161 306 | 597 | 1.00 (Ref) |  | 269 | 1.00 (Ref) |  |
| 2.0-2.9 servings/day | 216 | 157 007 | 562 | 0.92 (0.82-1.04) |  | 296 | 1.12 (0.95-1.32) |  |
| 3.0-3.9 servings/day | 250 | 82 657 | 301 | 0.93 (0.81-1.08) |  | 165 | 1.17 (0.96-1.43) |  |
| ≥4.0 servings/day | 292 | 64 201 | 244 | 1.01 (0.87-1.18) |  | 112 | 1.05 (0.84-1.31) |  |
| per 100 g/day |  |  |  | 0.98 (0.88-1.10) |  |  | 1.08 (0.93-1.26) |  |
|  |  |  |  | Ptrend=0.774 |  |  | Ptrend=0.328 | P=0.335 |
| Fibre |  |  |  |  |  |  |  |  |
| Lowest fifth | 12.9 | 91 213 | 319 | 1.00 (ref) |  | 168 | 1.00 (ref) |  |
| 2 | 14.9 | 90 766 | 326 | 0.97 (0.83-1.14) |  | 175 | 1.03 (0.83-1.28) |  |
| 3 | 16.2 | 90 396 | 329 | 0.97 (0.83-1.14) |  | 160 | 0.92 (0.74-1.15) |  |
| 4 | 17.4 | 90 481 | 331 | 0.96 (0.82-1.12) |  | 150 | 0.86 (0.69-1.08) |  |
| Highest fifth | 19.6 | 90 288 | 335 | 0.95 (0.81-1.11) |  | 164 | 0.91 (0.72-1.13) |  |
| per 5 g/day |  |  |  | 0.97 (0.87-1.08) |  |  | 0.90 (0.77-1.05) |  |
|  |  |  |  | Ptrend=0.578 |  |  | Ptrend=0.184 | P=0.445 |
| Alcohol |  |  |  |  |  |  |  |  |
| <1.0g/day | 2 | 92 768 | 318 | 1.00 (ref) |  | 126 | 1.00 (ref) |  |
| 1.0-7.9 g/day | 7 | 119 993 | 380 | 0.98 (0.84-1.14) |  | 179 | 1.12 (0.89-1.41) |  |
| 8.0-15.9 g/day | 15 | 104 849 | 371 | 1.07 (0.91-1.24) |  | 160 | 1.05 (0.82-1.33) |  |
| ≥16.0 g/day | 32 | 156 528 | 666 | 1.18 (1.02-1.36) |  | 399 | 1.40 (1.13-1.73) |  |
| per 10 g/day |  |  |  | 1.06 (1.02-1.11) |  |  | 1.11 (1.05-1.18) |  |
|  |  |  |  | Ptrend=0.004 |  |  | Ptrend<0.001 | P=0.218 |
| Tea |  |  |  |  |  |  |  |  |
| <2.0 cups/day | 147 | 124 710 | 431 | 1.00 (ref) |  | 227 | 1.00 (ref) |  |
| 2.0-3.9 cups/day | 460 | 139 179 | 553 | 1.07 (0.94-1.22) |  | 260 | 0.94 (0.79-1.13) |  |
| 4.0-5.9 cups/day | 679 | 120 345 | 437 | 0.97 (0.85-1.11) |  | 201 | 0.84 (0.69-1.01) |  |
| ≥6.0 cups/day | 880 | 89 260 | 310 | 0.94 (0.81-1.09) |  | 175 | 0.98 (0.80-1.19) |  |
| per 200 mL/day |  |  |  | 0.98 (0.94-1.02) |  |  | 0.98 (0.93-1.03) |  |
|  |  |  |  | Ptrend=0.281 |  |  | Ptrend=0.378 | P=0.921 |
| Coffee |  |  |  |  |  |  |  |  |
| 0 cups/day | 48 | 105 274 | 381 | 1.00 (ref) |  | 183 | 1.00 (ref) |  |
| 0.5-1.9 cup/day | 188 | 128 851 | 470 | 0.91 (0.79-1.04) |  | 224 | 0.89 (0.73-1.09) |  |
| 2.0-2.9 cups/day | 348 | 88 681 | 333 | 0.91 (0.78-1.06) |  | 176 | 0.98 (0.79-1.20) |  |
| ≥3.0 cups/day | 584 | 150 598 | 550 | 0.90 (0.79-1.03) |  | 277 | 0.90 (0.75-1.09) |  |
| per 200 mL/day |  |  |  | 0.97 (0.93-1.02) |  |  | 0.98 (0.91-1.04) |  |
|  |  |  |  | Ptrend=0.201 |  |  | Ptrend=0.484 | P=0.868 |
| CI: confidence interval HR: hazard ratio | | | | | | | | |
| ^*^Mean intake from participants who completed at least one 24-hour dietary assessment. For participants who completed more than one 24-hour dietary assessment, their mean intake was taken first, before taking the mean of the group | | | | | | | | |
| ^†^Fully adjusted model is stratified by age category (5 year categories), deprivation (Townsend score, quintiles), sex and region (10 regions), and adjusted for waist cirucmference (sex-specific quintiles), height (sex-specific quintiles), smoking (never, previous, current < 15 cigarettes/day, current 15 or more cigarettes per day, unknown), alcohol (<1 g/d, 1-7 g/d, 8-15 g/d, ≥16 g/d, unknown), education (College or University degree, vocational qualifications (other professional qualifications/NVQ or HND or HNC), optional national exams at ages 17 to 18 years (A levels/AS levels), national exams at age 16 years (O levels/GCSEs/CSEs), none of the above, unknown) , physical activity (low: < 10 excess MET-hours per week, moderate: 10-49.9 excess MET-hrs per week, high 50+ excess MET-hours per week, unknown) , family history of CRC (mother, father, or sibling with colorectal cancer), regular NSAID use (regular asprin or Ibuprofen use), regular Vit D supplement use, regular folate supplement use, and in women only: parity (0, 1-2, ≥3 live births, unknown), menopause status (pre-menopausal, post-menopausal, not sure - had a hysterectomy, not sure other reason, unknown), ever OCA use (never, ever, unknown), and ever HRT use (never, ever, unknown) | | | | | | | | |

| **Supplementary Table 9** Associations between dietary factors and proximal and distal colon cancer in UK Biobank | | | | | | | |  |
| --- | --- | --- | --- | --- | --- | --- | --- | --- |
| Reported consumption at recruitment | Mean intake (g/day)* | Participants | **Proximal colon** | |  | **Distal colon** | | Heterogeneity by subsite^‡^ |
|  |  |  | Cases | HR (95% CI) from fully adjusted model^†^ |  | Cases | HR (95% CI) from fully adjusted model^†^ |  |
| Red and processed meat |  |  |  |  |  |  |  |  |
| <2.0 times/week | 21 | 68 359 | 109 | 1.00 (Ref) |  | 64 | 1.00 (Ref) |  |
| 2.0-2.9 times/week | 52 | 135 973 | 265 | 1.03 (0.82-1.29) |  | 188 | 1.30 (0.98-1.73) |  |
| 3.0-3.9 times/week | 64 | 71 391 | 132 | 0.92 (0.71-1.20) |  | 126 | 1.56 (1.15-2.12) |  |
| ≥4.0 times/week | 76 | 192 600 | 359 | 0.91 (0.73-1.14) |  | 377 | 1.68 (1.28-2.21) |  |
| per 50 g/day |  |  |  | 0.90 (0.74-1.09) |  |  | 1.63 (1.29-2.05) |  |
|  |  |  |  | Ptrend=0.260 |  |  | Ptrend<0.001 | P<0.001 |
| Red meat |  |  |  |  |  |  |  |  |
| <1.0 time/week | 8 | 47 795 | 64 | 1.00 (Ref) |  | 52 | 1.00 (Ref) |  |
| 1.0-1.9 times/week | 34 | 184 816 | 338 | 1.12 (0.86-1.47) |  | 253 | 1.08 (0.80-1.46) |  |
| 2.0-2.9 times/week | 44 | 131 486 | 257 | 1.12 (0.84-1.47) |  | 238 | 1.31 (0.97-1.78) |  |
| ≥3.0 times/week | 54 | 104 813 | 206 | 1.06 (0.79-1.41) |  | 212 | 1.37 (1.00-1.87) |  |
| per 50 g/day |  |  |  | 1.02 (0.77-1.36) |  |  | 1.55 (1.13-2.13) |  |
|  |  |  |  | Ptrend=0.864 |  |  | Ptrend=0.006 | P=0.054 |
| Processed meat |  |  |  |  |  |  |  |  |
| Never | 5 | 44 107 | 67 | 1.00 (Ref) |  | 44 | 1.00 (Ref) |  |
| <1.0 time/week | 16 | 143 673 | 276 | 1.07 (0.82-1.41) |  | 204 | 1.24 (0.89-1.72) |  |
| 1.0 time/week | 22 | 138 239 | 263 | 1.02 (0.77-1.34) |  | 246 | 1.46 (1.05-2.03) |  |
| ≥2.0 times/week | 29 | 147 417 | 268 | 0.96 (0.73-1.27) |  | 268 | 1.44 (1.04-2.00) |  |
| per 25 g/day |  |  |  | 0.90 (0.70-1.16) |  |  | 1.41 (1.07-1.88) |  |
|  |  |  |  | Ptrend=0.408 |  |  | Ptrend=0.015 | P=0.019 |
| Poultry |  |  |  |  |  |  |  |  |
| Never | 2 | 24 328 | 35 | 1.00 (Ref) |  | 34 | 1.00 (Ref) |  |
| <1.0 time/week | 19 | 50 801 | 100 | 0.95 (0.64-1.40) |  | 82 | 0.83 (0.56-1.25) |  |
| 1.0/week | 28 | 169 686 | 326 | 0.96 (0.68-1.37) |  | 298 | 0.94 (0.66-1.35) |  |
| ≥2.0 times/week | 40 | 228 784 | 411 | 1.02 (0.72-1.45) |  | 349 | 0.92 (0.64-1.31) |  |
| per 25 g/day |  |  |  | 1.06 (0.89-1.27) |  |  | 1.00 (0.83-1.20) |  |
|  |  |  |  | Ptrend=0.511 |  |  | Ptrend=0.971 | P=0.632 |
| Total fish |  |  |  |  |  |  |  |  |
| <1 time/week | 6 | 36 512 | 63 | 1.00 (Ref) |  | 44 | 1.00 (Ref) |  |
| 1.0-1.9 times/week | 21 | 188 934 | 336 | 0.86 (0.66-1.13) |  | 306 | 1.13 (0.82-1.55) |  |
| 2.0-2.9 times/week | 29 | 111 375 | 204 | 0.80 (0.60-1.06) |  | 193 | 1.11 (0.79-1.54) |  |
| ≥3.0 times/week | 39 | 133 270 | 263 | 0.85 (0.65-1.13) |  | 214 | 1.00 (0.72-1.40) |  |
| per 25 g/day |  |  |  | 0.93 (0.77-1.11) |  |  | 0.93 (0.77-1.13) |  |
|  |  |  |  | Ptrend=0.405 |  |  | Ptrend=0.471 | P=0.967 |
| Dairy milk |  |  |  |  |  |  |  |  |
| never | 30 | 15 823 | 25 | 1.00 (Ref) |  | 29 | 1.00 (Ref) |  |
| <150 mL/day | 157 | 84 707 | 146 | 1.18 (0.77-1.80) |  | 134 | 0.90 (0.60-1.35) |  |
| 150-299 mL/day | 223 | 249 393 | 492 | 1.16 (0.77-1.73) |  | 432 | 0.88 (0.60-1.29) |  |
| 300 mL/day | 279 | 99 809 | 173 | 1.01 (0.67-1.55) |  | 138 | 0.70 (0.47-1.05) |  |
| per 100 mL/day |  |  |  | 0.96 (0.84-1.10) |  |  | 0.87 (0.76-0.99) |  |
|  |  |  |  | Ptrend=0.568 |  |  | Ptrend=0.036 | P=0.270 |
| Cheese |  |  |  |  |  |  |  |  |
| <1.0 time/week | 13 | 92 894 | 176 | 1.00 (ref) |  | 156 | 1.00 (ref) |  |
| 1.0 times/week | 18 | 99 265 | 176 | 0.91 (0.74-1.12) |  | 161 | 0.94 (0.75-1.17) |  |
| 2.0-4.9 times/week | 25 | 208 928 | 391 | 0.97 (0.81-1.16) |  | 316 | 0.87 (0.72-1.06) |  |
| ≥5.0 times/week | 39 | 61 039 | 112 | 1.02 (0.80-1.31) |  | 111 | 1.11 (0.86-1.42) |  |
| per 25 g/day |  |  |  | 1.05 (0.84-1.31) |  |  | 1.08 (0.85-1.37) |  |
|  |  |  |  | Ptrend=0.674 |  |  | Ptrend=0.551 | P=0.883 |
| Fruit |  |  |  |  |  |  |  |  |
| <2.0 servings/day | 107 | 154 678 | 280 | 1.00 (Ref) |  | 251 | 1.00 (Ref) |  |
| 2.0-2.9 servings/day | 181 | 118 743 | 202 | 0.91 (0.76-1.09) |  | 180 | 0.93 (0.77-1.13) |  |
| 3.0-3.9 servings/day | 231 | 90 574 | 168 | 0.97 (0.80-1.19) |  | 147 | 0.99 (0.81-1.22) |  |
| ≥4.0 servings/day | 310 | 103 966 | 212 | 1.04 (0.86-1.25) |  | 169 | 0.98 (0.80-1.20) |  |
| per 100 g/day |  |  |  | 1.02 (0.93-1.12) |  |  | 0.99 (0.90-1.10) |  |
|  |  |  |  | Ptrend=0.654 |  |  | Ptrend=0.910 | P=0.698 |
| Vegetables |  |  |  |  |  |  |  |  |
| <2.0 servings/day | 162 | 161 306 | 280 | 1.00 (Ref) |  | 251 | 1.00 (Ref) |  |
| 2.0-2.9 servings/day | 216 | 157 007 | 294 | 1.03 (0.87-1.21) |  | 180 | 0.84 (0.70-1.00) |  |
| 3.0-3.9 servings/day | 250 | 82 657 | 157 | 1.04 (0.85-1.27) |  | 147 | 0.85 (0.68-1.05) |  |
| ≥4.0 servings/day | 292 | 64 201 | 126 | 1.13 (0.91-1.40) |  | 169 | 0.92 (0.73-1.15) |  |
| per 100 g/day |  |  |  | 1.08 (0.93-1.26) |  |  | 0.90 (0.76-1.06) |  |
|  |  |  |  | Ptrend=0.311 |  |  | Ptrend=0.193 | P=0.100 |
| Fibre |  |  |  |  |  |  |  |  |
| Lowest fifth | 12.9 | 91 213 | 162 | 1.00 (ref) |  | 134 | 1.00 (ref) |  |
| 2 | 14.9 | 90 766 | 173 | 1.01 (0.81-1.25) |  | 143 | 1.03 (0.81-1.31) |  |
| 3 | 16.2 | 90 396 | 168 | 0.97 (0.78-1.21) |  | 139 | 1.00 (0.78-1.27) |  |
| 4 | 17.4 | 90 481 | 152 | 0.85 (0.68-1.07) |  | 160 | 1.13 (0.89-1.44) |  |
| Highest fifth | 19.6 | 90 288 | 178 | 0.98 (0.78-1.22) |  | 138 | 0.97 (0.75-1.24) |  |
| per 5 g/day |  |  |  | 0.95 (0.81-1.11) |  |  | 1.00 (0.84-1.18) |  |
|  |  |  |  | Ptrend=0.534 |  |  | Ptrend=0.974 | P=0.690 |
| Alcohol |  |  |  |  |  |  |  |  |
| <1.0g/day | 2 | 92 768 | 169 | 1.00 (ref) |  | 133 | 1.00 (ref) |  |
| 1.0-7.9 g/day | 7 | 119 993 | 198 | 0.98 (0.79-1.20) |  | 156 | 0.95 (0.75-1.20) |  |
| 8.0-15.9 g/day | 15 | 104 849 | 208 | 1.14 (0.92-1.40) |  | 148 | 1.00 (0.79-1.27) |  |
| ≥16.0 g/day | 32 | 156 528 | 299 | 1.00 (0.82-1.23) |  | 325 | 1.32 (1.06-1.64) |  |
| per 10 g/day |  |  |  | 1.00 (0.94-1.06) |  |  | 1.12 (1.05-1.19) |  |
|  |  |  |  | Ptrend=0.950 |  |  | Ptrend<0.001 | P=0.012 |
| Tea |  |  |  |  |  |  |  |  |
| <2.0 cups/day | 147 | 124 710 | 225 | 1.00 (ref) |  | 179 | 1.00 (ref) |  |
| 2.0-3.9 cups/day | 460 | 139 179 | 266 | 0.99 (0.83-1.18) |  | 265 | 1.23 (1.02-1.49) |  |
| 4.0-5.9 cups/day | 679 | 120 345 | 215 | 0.91 (0.75-1.10) |  | 194 | 1.03 (0.84-1.27) |  |
| ≥6.0 cups/day | 880 | 89 260 | 166 | 0.97 (0.79-1.18) |  | 123 | 0.89 (0.71-1.12) |  |
| per 200 mL/day |  |  |  | 0.98 (0.93-1.03) |  |  | 0.97 (0.92-1.03) |  |
|  |  |  |  | Ptrend=0.490 |  |  | Ptrend=0.299 | P=0.774 |
| Coffee |  |  |  |  |  |  |  |  |
| 0 cups/day | 48 | 105 274 | 182 | 1.00 (ref) |  | 172 | 1.00 (ref) |  |
| 0.5-1.9 cup/day | 188 | 128 851 | 230 | 0.92 (0.76-1.12) |  | 207 | 0.90 (0.73-1.11) |  |
| 2.0-2.9 cups/day | 348 | 88 681 | 178 | 1.00 (0.81-1.24) |  | 140 | 0.86 (0.69-1.08) |  |
| ≥3.0 cups/day | 584 | 150 598 | 285 | 0.96 (0.79-1.16) |  | 242 | 0.88 (0.71-1.07) |  |
| per 200 mL/day |  |  |  | 1.00 (0.93-1.06) |  |  | 0.96 (0.90-1.03) |  |
|  |  |  |  | Ptrend=0.931 |  |  | Ptrend=0.272 | P=0.457 |
| CI: confidence interval HR: hazard ratio | | | | | | | | |
| ^*^Mean intake from participants who completed at least one 24-hour dietary assessment. For participants who completed more than one 24-hour dietary assessment, their mean intake was taken first, before taking the mean of the group | | | | | | | | |
| ^†^Fully adjusted model is stratified by age category (5 year categories), deprivation (Townsend score, quintiles), sex and region (10 regions), and adjusted for waist circumference (sex-specific quintiles), height (sex-specific quintiles), smoking (never, previous, current < 15 cigarettes/day, current 15 or more cigarettes per day, unknown), alcohol (<1 g/d, 1-7 g/d, 8-15 g/d, ≥16 g/d, unknown), education (College or University degree, vocational qualifications (other professional qualifications/NVQ or HND or HNC), optional national exams at ages 17 to 18 years (A levels/AS levels), national exams at age 16 years (O levels/GCSEs/CSEs), none of the above, unknown) , physical activity (low: < 10 excess MET-hours per week, moderate: 10-49.9 excess MET-hrs per week, high 50+ excess MET-hours per week, unknown) , family history of CRC (mother, father, or sibling with colorectal cancer), regular NSAID use (regular asprin or Ibuprofen use), regular Vit D supplement use, regular folate supplement use, and in women only: parity (0, 1-2, ≥3 live births, unknown), menopause status (pre-menopausal, post-menopausal, not sure - had a hysterectomy, not sure other reason, unknown), ever OCA use (never, ever, unknown), and ever HRT use (never, ever, unknown) | | | | | | | | |

| **Supplementary Table 10** Associations between alcohol types and colon and rectal cancer in UK Biobank | | | | | | | |  |
| --- | --- | --- | --- | --- | --- | --- | --- | --- |
| Reported consumption at recruitment | Mean intake (g/day)* | Participants | **Colon** | |  | **Rectal** | | Heterogeneity by subsite^‡^ |
|  |  |  | Cases | HR (95% CI) from fully adjusted model^†^ |  | Cases | HR (95% CI) from fully adjusted model^†^ |  |
| Alcohol from beer |  |  |  |  |  |  |  |  |
| < 1.0g/day | 0.9 | 174 007 | 619 | 1.00 (ref) |  | 265 | 1.00 (ref) |  |
| 1.0-7 .9g/day | 4.3 | 76 605 | 281 | 1.02 (0.87-1.19) |  | 154 | 1.04 (0.83-1.29) |  |
| 8.0-15.9 g/day | 10.5 | 41 992 | 160 | 0.98 (0.81-1.19) |  | 87 | 0.92 (0.70-1.20) |  |
| ≥16.0 g/day | 25.8 | 55 529 | 279 | 1.22 (1.03-1.46) |  | 196 | 1.43 (1.13-1.80) |  |
| Per 100 g/day |  |  |  | 1.09 (1.02-1.16) |  |  | 1.16 (1.07-1.27) |  |
|  |  |  |  | Ptrend=0.016 |  |  | Ptrend=0.001 | P=0.223 |
| Alcohol from wine |  |  |  |  |  |  |  |  |
| < 1.0g/day | 1.6 | 80 984 | 299 | 1.00 (ref) |  | 181 | 1.00 (ref) |  |
| 1.0-7 .9g/day | 6.8 | 121 189 | 439 | 0.99 (0.85-1.16) |  | 224 | 0.88 (0.71-1.08) |  |
| 8.0-15.9 g/day | 14.8 | 84 833 | 333 | 1.05 (0.89-1.24) |  | 144 | 0.78 (0.62-0.99) |  |
| ≥16.0 g/day | 28.4 | 59 197 | 259 | 1.14 (0.95-1.35) |  | 148 | 1.06 (0.84-1.34) |  |
| Per 100 g/day |  |  |  | 1.05 (0.99-1.12) |  |  | 1.03 (0.95-1.12) |  |
|  |  |  |  | Ptrend=0.083 |  |  | Ptrend=0.443 | P=0.689 |
| Alcohol from spirits |  |  |  |  |  |  |  |  |
| < 1.0g/day | 0.3 | 228 149 | 807 | 1.00 (ref) |  | 436 | 1.00 (ref) |  |
| 1.0-7 .9g/day | 1.5 | 91 095 | 357 | 0.95 (0.84-1.07) |  | 187 | 0.92 (0.77-1.09) |  |
| 8.0-15.9 g/day | 4.7 | 16 944 | 96 | 1.17 (0.95-1.46) |  | 46 | 1.03 (0.75-1.40) |  |
| ≥16.0 g/day | 11 | 10 606 | 60 | 1.07 (0.82-1.40) |  | 32 | 1.01 (0.70-1.46) |  |
| Per 10 g/day |  |  |  | 1.12 (0.89-1.42) |  |  | 1.02 (0.73-1.40) |  |
|  |  |  |  | Ptrend=0.336 |  |  | Ptrend=0.927 | P=0.626 |
| CI: confidence interval HR: hazard ratio | | | | | | | | |
| ^*^Mean intake from participants who completed at least one 24-hour dietary assessment. For participants who completed more than one 24-hour dietary assessment, their mean intake was taken first, before taking the mean of the group | | | | | | | | |
| ^†^Fully adjusted model is stratified by age category (5 year categories), deprivation (Townsend score, quintiles), sex and region (10 regions), and adjusted for waist cirumference (sex-specific quintiles), height (sex-specific quintiles), smoking (never, previous, current < 15 cigarettes/day, current 15 or more cigarettes per day, unknown), alcohol (<1 g/d, 1-7 g/d, 8-15 g/d, ≥16 g/d, unknown), education (College or University degree, vocational qualifications (other professional qualifications/NVQ or HND or HNC), optional national exams at ages 17 to 18 years (A levels/AS levels), national exams at age 16 years (O levels/GCSEs/CSEs), none of the above, unknown) , physical activity (low: < 10 excess MET-hours per week, moderate: 10-49.9 excess MET-hrs per week, high 50+ excess MET-hours per week, unknown) , family history of CRC (mother, father, or sibling with colorectal cancer), regular NSAID use (regular asprin or Ibuprofen use), regular Vit D supplement use, regular folate supplement use, and in women only: parity (0, 1-2, ≥3 live births, unknown), menopause status (pre-menopausal, post-menopausal, not sure - had a hysterectomy, not sure other reason, unknown), ever OCA use (never, ever, unknown), and ever HRT use (never, ever, unknown) | | | | | | | | |
